# A Role for Id2 in Regulating Photic Entrainment of the Mammalian Circadian System

**DOI:** 10.1016/j.cub.2008.12.052

**Published:** 2009-02-24

**Authors:** Giles E. Duffield, Nathan P. Watson, Akio Mantani, Stuart N. Peirson, Maricela Robles-Murguia, Jennifer J. Loros, Mark A. Israel, Jay C. Dunlap

**Affiliations:** 1 Department of Genetics, Dartmouth Medical School, Hanover, NH 03755, USA; 2 Department of Biochemistry, Dartmouth Medical School, Hanover, NH 03755, USA; 3 Norris Cotton Cancer Center & Department of Pediatrics, Rubin Building, 1 Medical Center Drive, Lebanon, NH 03756, USA; 4 Department of Biological Sciences, Galvin Life Science Center, University of Notre Dame, Notre Dame, IN 46556, USA; 5 Nuffield Laboratory of Ophthalmology, University of Oxford, The John Radcliffe Hospital, Headley Way, Oxford OX3 9DU, UK

**Keywords:** DNA, MOLNEURO

## Abstract

*Inhibitor of DNA binding* genes (*Id1–Id4*) encode helix-loop-helix (HLH) transcriptional repressors associated with development and tumorigenesis [1, 2], but little is known concerning the function(s) of these genes in normal adult animals. *Id2* was identified in DNA microarray screens for rhythmically expressed genes [3–5], and further analysis revealed a circadian pattern of expression of all four *Id* genes in multiple tissues including the suprachiasmatic nucleus. To explore an in vivo function, we generated and characterized deletion mutations of *Id2* and of *Id4*. *Id2*^−/−^ mice exhibit abnormally rapid entrainment and an increase in the magnitude of the phase shift of the pacemaker. A significant proportion of mice also exhibit disrupted rhythms when maintained under constant darkness. Conversely, *Id4*^−/−^ mice did not exhibit a noticeable circadian phenotype. In vitro studies using an *mPer1* and an *AVP* promoter reporter revealed the potential for ID1, ID2, and ID3 proteins to interact with the canonical basic HLH clock proteins BMAL1 and CLOCK. These data suggest that the *Id* genes may be important for entrainment and operation of the mammalian circadian system, potentially acting through BMAL1 and CLOCK targets.

## Results and Discussion

Many aspects of biochemistry, physiology, and behavior are organized around a 24 hr rhythm, driven by an endogenous circadian clock [6]. Interlocked autoregulatory molecular transcriptional-translational feedback loops underlie circadian organization in single cells. Two core feedback loops have been described in the mammal; they consist of two *period* (*mPer*) genes and two *cryptochrome* (*mCry*) genes (negative loop), and basic helix-loop-helix/Per-Arnt-Sim (bHLH/PAS) domain proteins BMAL1 (MOP3) and CLOCK (positive loop) [7, 8]. The master circadian oscillator in the mammal is located within the hypothalamic suprachiasmatic nucleus (SCN) [7], although many tissues of the body, as well as cell lines derived from them, harbor a circadian clock [9–11]. cDNA microarray analysis of one such line of mammalian fibroblasts identified a series of rhythmically expressed genes with a circadian periodicity [3]. Among these genes was *inhibitor of DNA binding 2* (*Id2*) (Figure S1 available online), a member of an HLH transcriptional repressor family previously implicated in the regulation of differentiation, cell cycle, and apoptosis, primarily during early development, and whose misregulation is associated with tumorigenesis [1, 2, 12].

*Id2* is a member of a family of four genes, *Id1*–*Id4*, that share both structural and functional properties [1, 12, 13]. At the molecular level, it is known that ID proteins interact with bHLH factors through their HLH protein-protein binding domains; however, because they do not possess the basic region, the ID proteins sequester the bound bHLH transcription factor and prevent its binding to its partner bHLH factor. This, in turn, inhibits binding of the heterodimer to DNA. Given the identity of CLOCK and BMAL1 as bHLH transcription factors, the potential for ID proteins to influence the circadian system seemed apparent.

### *Id* Genes Are Rhythmically Expressed in Tissues Harboring Central and Peripheral Clocks

**Figure 1:**
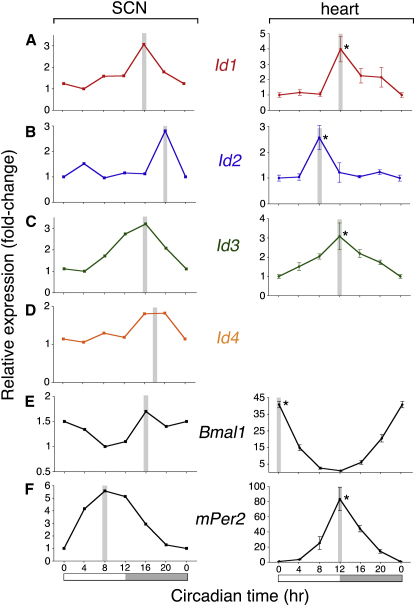
*Id* Genes Are Rhythmically Expressed in the SCN and Heart (A–F) The following are shown: A, *Id1*; B, *Id2*; C, *Id3*; D, *Id4* (only expressed in SCN); E, *Bmal1*; and F, *mPer2*. SCN profiles are left and heart profiles are right. Mouse tissue was collected under constant dark conditions (3 days after transition from 12:12 LD cycle to DD). SCN samples were collected as pooled frozen tissue punches (n = 6 mice per time point). Heart samples were analyzed for each animal (n = 3–6 mice per time point). Quantitative gene expression was measured by real-time quantitative RT-PCR (SYBR green, ABI 7700, normalized to 18S and *acidic ribosomal phosphoprotein*, *ARP*). Values are *n*-fold-change relative to nadir of expression and mean ± SEM for heart. The peak phase of rhythm is indicated by the gray bar. Subjective day and night are highlighted by white and gray panels below (F), respectively. Statistical analysis of heart samples reveals significant rhythms (one-factor ANOVA, followed by Dunnett's post-hoc t tests, ^∗^p < 0.05).

Circadian regulation of *Id* genes was determined in SCN and heart tissue collected from mice held in constant darkness (DD) (Figure 1). In the SCN, expression of all four *Id* genes was rhythmic, with peak phases occurring near the middle of subjective night at circadian time (CT) 16 to CT20. In the heart, peak phases of *Id1*, *Id2*, and *Id3* span the middle to late subjective day, CT8 to CT12, whereas expression of *Id4* was below the level of detection. Impressively, *Id* gene expression showed a distinct circadian pattern in all tissues examined, namely SCN, heart, liver (peak phase at CT4; T. Hou and G.E.D., data not shown), and fibroblasts.

The phases of rhythms of known clock genes, such as *mPer1–2*, *mCry1–2*, and *Bmal1*, are phase locked to one another in a characteristic pattern within and between individual tissues [6, 7]. For instance, *mPer1* peaks ∼2–4 hr prior to *mPer2*, both are antiphasic to *Bmal1*, and all these phases are delayed by 3–9 hr in peripheral tissues as compared to the SCN [6, 7] (Figure 1). This characteristic pattern is seen for *Id2*, whose peak phase lags that of *Bmal1* in SCN, heart, liver, and rat1 and mouse NIH 3T3 fibroblasts by approximately 4–8 hr, although not for other *Id* genes (Figure 1 and Figure S1).

### *Id2*^−/−^ Mice Exhibit Disrupted Locomotor Rhythms, Reduced Locomotor Activity, and a Change in Phase Angle

**Figure 2:**
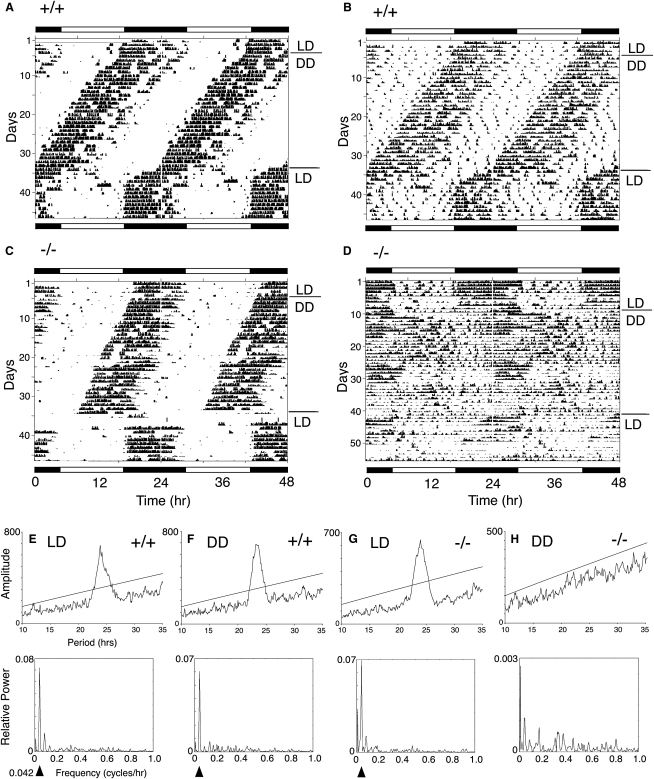
A Proportion of *Id2* Mutant Mice Show Severe Disruption of Locomotor Activity Rhythms (A–D) Activity records of representative wild-type (+/+; [A] and [B]) and *Id2* mutant (−/−; [C] and [D]) mice are shown in double-plotted format. Each horizontal line represents a 48 hr period, and the second 24 hr period is plotted to the right and below the first. Vertical bars represent periods of wheel-running activity (A and C) or general activity as measured by a passive infrared detector (B and D). Animals were individually housed in a 12:12 LD cycle for at least 14 days, transferred to DD for 30 days, and then transferred back to a LD cycle. The line above DD on the right indicates the transition from LD to DD. The timing of the respective light-dark cycles is indicated by the white-and-black bars above and below the records. Numbers on the left indicate the number of days in the study. Wild-type mice maintained robust rhythmicity in both LD and DD, as is reflected by the representative mice (A and B). Of the *Id2* mutant mice, 75% showed robust to distinct rhythms in both LD and DD, as shown in the representative animal (C), whereas 25% became arrhythmic or showed very weak rhythmicity in DD, and in some cases in LD, as shown in the representative animal (D); see also Figure S4. Note that the animal in (B) was not provided with a running wheel, and that mouse (D) activity data were collected for a longer duration. (E–H) χ^2^ periodogram and Fourier analysis of the locomotor activity rhythms. Plots represent analysis of 10 days of data from a representative wild-type mouse in LD (E) and DD (F) and from an *Id2* mutant mouse in LD (G) and DD (H). Analyses are for the mice whose actograms are as shown in (B) and (D). The analyses were undertaken on the last 10 day duration in LD and on the third 10 day duration in DD. For the periodogram analysis, the ascending straight line represents a statistical significance of p = 0.001. For Fourier analysis, a frequency of 0.042 cycles/hr corresponds to 1 cycle/24 hr, which is denoted by the arrowhead below the chart where statistically significant (statistical significance was determined by the Clocklab program). Note the changes in the y axis between charts, and a reduction in values, indicates a loss of rhythm amplitude in DD. In LD, all wild-type and *Id2*^+/−^ mice had a significant rhythm with a period length of 23.9–24.0 hr and showed significant rhythms in DD with variable period lengths. In these example mice, the wild-type mouse had a significant rhythm in both LD and in DD, with period lengths of 24.0 hr and 23.5 hr, respectively. In the example *Id2* mutant mouse, a significant rhythm was detected in LD but no significant rhythm was detected in DD. Also note that the rhythm in LD was robust in days 1–10 (G), but after the duration in DD, in the LD during days 44–57, only a weak rhythm was identified (analysis not shown: periodogram, n.s.; Fourier analysis, significant peak at ∼0.042 cycles/hr of 0.02).

Because *Id2* had shown the most consistent rhythmic gene expression in all cell lines and tissues, we generated transgenic mice lacking the entire coding region and therefore null for the *Id2* gene (Figure S2 and Supplemental Discussion) and examined their circadian systems (Figure 2, Table S1). Homozygous mutants are morphologically dissimilar to their wild-type littermate controls, being on average 27% smaller (F_2,56_ = 9.1, p = 0.0004), and exhibit a high level of lethality during the postnatal period; our other broad observations of the *Id2*^−/−^ mice, such as immunocompromised status, are consistent with the *Id2* null mice generated by Yokota and colleagues [14] (see Supplemental Data). Importantly, histological analysis of SCN coronal sections showed no gross anatomical difference between *Id*2^+/+^ and *Id2*^−/−^ F2 littermates (Figure S3), indicating that any circadian phenotypes in the mutants are not due to a gross developmental defect in the basic organization of the SCN.

Animals of all three genotypes were capable of entrainment and free-running rhythmicity, but a significant fraction (25%) of the homozygous mutant mice showed severely disrupted circadian rhythms (Figures 2D, 2G, and 2H and Figure S4), thereby defining a significant phenotype (Fisher's Exact test, df = 2,44, p = 0.0284; Figures S4–S6). They showed reduced use of the running wheel coupled with disorganized rhythms, as determined by analysis of *passive activity* (as measured by passive infrared motion detector; Figure 2D and Figure S4), with no significant rhythmicity detected during the last 10 days in DD (Figure 2H and Figure S6). Several additional older mice (>6 months age) developed antiphase diurnal rhythms while maintaining appropriate phase-response curves (data not shown). *Id2* mutant mice with disrupted rhythms remained indistinguishable from the remaining *Id2*^−/−^ mice as regards weight, gross morphology, and general health.

The entire class of *Id2*^−/−^ mice displayed a 70% reduction in wheel-running activity as measured by mean wheel rotations/24 hr (F_2,37_ = 17.0, p = 0.0001; light-dark [LD], mean ± standard error of the mean [SEM]: wild-type, 23,674 ± 1,278; heterozygote, 23,846 ± 3,045; and mutant, 7,687 ± 2,154). This is also reflected in the *power* measure at 24 hr derived from the Fourier analysis of wheel-running activity, a measure of amplitude of the detected rhythm, which is significantly lower in the mutant mice in both LD and DD (LD, F_2,38_ = 4.5, p = 0.0179; DD, F_2,32_ = 3.7, p = 0.037; Table S1) even when the four arrhythmic mice are excluded from the analysis. Their arrhythmic passive activity cannot be explained by the low quantity of wheel-running activity, because the passive activity of wild-type and heterozygote controls, tested in the absence of a running wheel, exhibited robust rhythms (Figure 2B). Moreover, certain *Id2*^−/−^ mice exhibited clear rhythmicity of both wheel-running and passive activity despite showing little daily use of the wheel (Figures S4A, S4F, and S5A). No difference was observed between mutant mice and littermate controls in the quantity of their passive daily activity (Table S1; LD, mean ± SEM counts: wild-type, 2,878 ± 431; heterozygote, 3,100 ± 796; and mutant, 3,358 ± 374) nor in the *power* measure for passive activity in either LD or DD (Table S1). It seems that, for whatever reason, mice lacking *Id2* can be characterized by reduced running on a wheel independent of their apparent circadian defects.

Free-running periods of the experimental mice were similar (wild-type, 23.78 ± 0.10 hr; heterozygote; 23.62 ± 0.05 hr; and mutant, 23.89 ± 0.10 hr; mean ± SEM), as were several other aspects of their circadian rhythmicity (Table S1). Interestingly, however, the phase angle of activity onset relative to the time of lights off (ZT12) was significantly different in mutant mice (t_1,24_ = 2.3, p < 0.05; Figure S7 and Table S1): all but one out of the 13 knockout mice showed a phase angle ≤ +30 min (that is, 30 min before ZT12), whereas six of the 13 wild-type mice exhibited phase angles between +36 and +106 min (mean ± SEM phase angles of +24 ± 11 min, +40 ± 21 min, and −3 ± 7 min for *Id2*^+/+^, *Id2*^+/−^, and *Id2*^−/−^ mice, respectively). In addition, the variance of the phase angle was smaller in the mutant mice (wild-type, 1504; heterozygote, 5299; and mutant mice, 680). Thus, loss of ID2 can significantly affect entrainment as discussed more fully below.

### Enhanced Speed of Re-entrainment and Magnitude of Phase Shifts in *Id2*^−/−^ Mice

**Figure 3:**
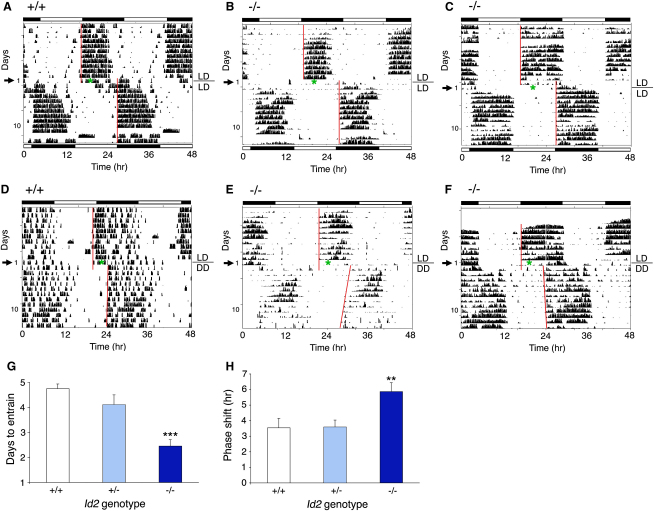
*Id2* Mutant Mice Show Abnormally Rapid Entrainment to a New Light-Dark Cycle (A–F) Representative locomotor activity records of wild-type (A and D) and *Id2* mutant (B, C, E, and F) exposed to a 10 hr extension of the light phase of the LD cycle. Mice were maintained on a LD cycle for at least 14 days and transferred to a new cycle where ZT12 is delayed by 10 hr by extending the light phase of the LD cycle by 10 hr on day 1 (A–C). In (D)–(F), mice were exposed to the equivalent treatment of 10 hr prolonged light exposure on day 1 and then transferred to DD for the rest of the experiment. The line above DD on the right indicates the transition from LD to DD. The timing of the respective LD cycles is indicated by the white-and-black bars above and below the records. Numbers on the left indicate the number of days following the transition to the new LD cycle or the 10 hr light treatment prior to transfer to DD. The arrow on the left indicates the actual day of treatment (day 1), and the actual midtime of treatment is marked by a green asterisk within the actogram. A red line is fitted to the phase of activity onset for several days before and after the shift of the LD cycle or transfer to DD. (G) Numbers of days required for stable entrainment to the new photoschedule are shown in the histogram for wild-type (white), heterozygote (pale blue), and mutant (dark blue) mice (mean ± SEM). (H) Mean ± SEM magnitude of the phase shifts produced by light treatment is shown for wild-type (white), heterozygote (pale blue), and mutant (dark blue) mice. Extrapolated activity onsets of the first day after the 10 hr light treatment were used to determine the size of resultant phase delays. The values marked by asterisks are statistically significant as determined by one-factor ANOVA (Dunnett's post-hoc t tests, ^∗∗^p < 0.01, ^∗∗∗^p < 0.001).

To examine the effect of the mutation on photic entrainment in greater detail, we measured the re-entrainment response of mice to a large delay in the daily photoschedule. Normally, mice entrained to a 12:12 LD cycle became active around the transition to lights-off, and after a 10 hr delay of this photoschedule, their activity-rest cycle achieved stable re-entrainment after 4.75 ± 0.19 days (wild-types) or 4.11 ± 0.39 days (heterozygotes). Surprisingly, however, the mutant mice showed a highly significant more rapid re-entrainment in 2.46 ± 0.26 days (F_2,27_ = 18.8, p = 0.0001, day 1 = day of treatment, Figures 3A–3C and 3G).

To remove the possibility that this unexpected phenotype reflects a masking response due to the repeated light-dark cycle, we repeated the experiment with a modified protocol in which mice were transferred to DD after a single long day representing the shift of the photoschedule. The magnitude of the shift produced by this single 10 hr extension of the light phase was 3.54 ± 0.60 hr in wild-types and 3.59 ± 0.45 hr in heterozygotes, whereas consistent with the re-entrainment data, the phase shift of the mutant mice was significantly greater, at 5.87 ± 0.57 hr (F_2,31_ = 5.8, p = 0.0071; Figures 3D–3F and 3H).

In circadian theory, the effects of entraining agents have been modeled in two ways. In one, discrete or *nonparametric* entrainment, the phase of the circadian oscillator is thought to be abruptly shifted to the new phase soon after exposure to the entraining agent; in the other, continuous or *parametric* entrainment, the entraining agent acts continuously to adjust the period length of the oscillator so that rhythms in the exposed and unexposed organisms gradually move out of phase [15]. By this formalism, the 10 hr delay re-entrainment task could have yielded shifts by either mechanism, but the two can be distinguished through the use of short discrete pulses of light. Thus, to better characterize the role of *Id2* on photoentrainment, we examined mice free running in DD after exposure to a discrete 30 min light pulse at CT14 or CT16, times expected to produce a large phase delay [16]. Surprisingly, unlike the large phase delays observed in *Id2*^−/−^ mice after the continuous 10 hr light treatment, no difference was observed between genotypes when this discrete pulse of light was used (CT14, F_2,32_ = 2.24, n.s; CT16, t_1,26_ = 0.70, n.s.; Figure S8 and Table S1). These results indicate that the resetting mechanisms underlying continuous versus discrete entrainment are differentially influenced by ID2 (but not ID4; see below). We then examined the locomotor activity of mice under constant-light conditions. Similar proportions of mice exhibited a loss of a discernible locomotor rhythm, as determined by Fourier and χ^2^ periodogram analyses: *Id2*^+/+^ 38%, *Id2*^+/−^, 33%; and *Id2*^−/−^, 40%; no significant difference was observed between genotypes in free-running period lengths (Table S1). All mice showed an increase in period length with an increase in illuminance (p < 0.01), as predicted by Aschoff's rule [15].

In light of the photoentrainment phenotype, we examined the induction of *Id* genes in the SCN after light treatment during subjective night. Positive control genes *c-fos* and *mPer2* were normally induced, but we found no increase in levels of *Id1*–*4* expression (Figure S9).

### Suppression of CLOCK:BMAL1 Transactivation by ID Proteins

**Figure 4:**
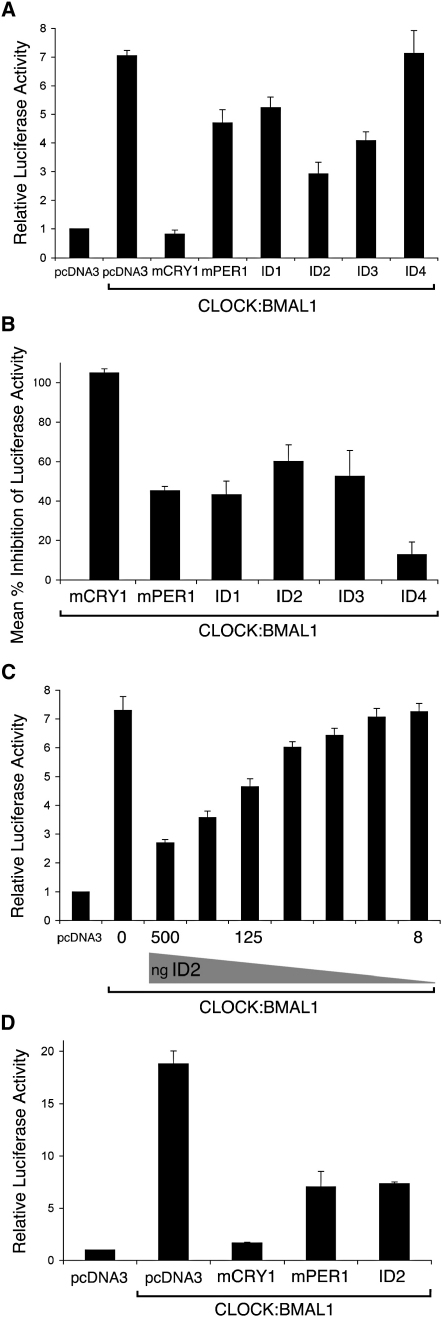
ID Proteins Can Potently Inhibit CLOCK:BMAL1 Transactivation of the *mPer1* and *AVP* Promoters (A) Effect of cotransfection of CLOCK and BMAL1 with ID constructs on transactivation of the *mPer1*-promoter. Data are mean ± SEM (n = 3–6) relative luciferase activity. All groups except pcDNA3.1 (empty vector) were transfected with equal amounts of CLOCK and BMAL1 expression vectors. As expected, luciferase activity was increased by the presence of plasmids encoding CLOCK and BMAL1, but not by either CLOCK or BMAL1 alone. Level of inhibition by clock proteins mCRY1 and mPER1 was similar to published reports [18, 37]. (B) Summary of five independent experiments showing consistent inhibition of CLOCK:BMAL1 transactivation at the E-box of the *mPer1*-promoter by ID1 (43%), ID2 (60%), and ID3 (53%) at levels similar or greater than shown by the clock protein mPER1. ID4 showed limited inhibitory activity. Values are mean ± SEM percentage inhibition of luciferase activity. (C) Dose-dependent repression of CLOCK:BMAL1 activity by ID2. Serial dilution of ID2 expression plasmid cotransfected with CLOCK and BMAL1 plasmids is shown. (D) Effect of cotransfection of CLOCK and BMAL1 with an ID2 expression plasmid on transactivation of the *arginine vasopressin* (*AVP*)-promoter. Data are mean ± SEM (n = 3) relative luciferase activity from one representative experiment. The amount of inhibition by ID2 is similar to that of mPER1. Level of inhibition by mPER1 was similar to published reports [18, 37].

As outlined above, the ID proteins are well known as repressors of the function of bHLH proteins, the class of protein to which CLOCK and BMAL1 belong. Because this suggested a potential mechanism by which the ID proteins might affect the clock, we examined the effect of each ID protein upon CLOCK:BMAL1-driven induction of *mPer1* gene activity. In this transient transfection assay, ID1, ID2, or ID3 showed marked dose-dependent suppression of CLOCK:BMAL1-induced activation of *mPer1*, with the ID proteins showing a repression activity equal to or greater than that of mPER1 itself (Figures 4A–4C). However, little inhibitory effect was observed for ID4, a finding confirmed with an alternative expression vector for ID4 (data not shown). Because the ID proteins repress bHLH proteins through inhibition of DNA binding, we examined the ability of ID2 to suppress E-box-driven CLOCK:BMAL1 transactivation of the arginine vasopressin (*AVP*) gene [7, 17]. The single E-box is the only region conserved between the *mPer1* and *AVP* promoters [18]. Consistent with the results for the *mPer1* reporter, ID2 suppressed the *AVP* reporter activity by an average of 64%, similar to that demonstrated by mPER1 (Figure 4D) and consistent with ID inhibition of transcription through interference with CLOCK:BMAL1 transactivation of the E-box element. The specificity of the inhibitory action of ID1, ID2, and ID3, but not ID4, is particularly interesting and suggests that the interaction between ID1-3 and CLOCK:BMAL1 is not simply a generalized action on HLH domains.

### *Id4*^−/−^ Mice Exhibit Normal Entrainment Responses

To explore the difference observed in the inhibitory action of ID2 versus ID4 upon canonical clock components, we analyzed the behavior of *Id4* null mice and their wild-type and heterozygous littermates in vivo [19]. Apart from a reduction in wheel-running activity, most *Id4*^−/−^ mice exhibited normal rhythmic profiles, as determined by actogram, periodogram, and Fourier analysis (data not shown). Because of the profound entrainment phenotype observed in *Id2*^−/−^ mice, we measured the magnitude of the phase shift of *Id4*^−/−^ mice in response to a 10 hr extension of the light phase followed by transfer to DD. The shift in the phase of activity onset was 3.79 ± 0.18 hr in wild-type mice, 4.20 ± 0.24 hr in heterozygote mice, and 3.50 ± 0.09 hr in *Id4*^−/−^ mice. Therefore, unlike the findings for *Id2*^−/−^ mice, no significant difference was observed in the shift in the phase of activity onset of the *Id4* mutant mice (F_2,24_ = 2.9, n.s.; Figure S10).

The *Id* genes have well-characterized roles in embryonic differentiation and cell proliferation as well as in the differentiation of immune cells, but few other roles in normal adult tissues have been described. As revealed by our microarray and real-time RT-PCR analyses, *Id2* is rhythmically expressed in mammalian fibroblasts and liver. Subsequent examination of murine SCN and heart in vivo revealed robust rhythmic patterns of expression for all four family members, where expressed. Our findings are consistent with the discovery of *Id* genes as rhythmically expressed in various tissues, specifically *Id1* in the pineal gland and immortalized 2.2 SCN cells [20, 21], *Id2* in SCN and liver [4, 5], and *Id3* in the SCN [5, 21] (see Supplemental Discussion). What is particularly striking is the conserved phase-specific relationship for the *Id2* rhythm with the canonical clock genes in all tissues examined, consistent with a possible effect of *Id2* upon the circadian system. Apart from the canonical clock components, this is rare for rhythmic genes: less than 10% of rhythmic genes are found in common between two different tissue types (e.g., SCN and liver) [4, 5, 22, 23]. These results suggested the significance of ID2 for circadian biology.

Consistent with this hypothesis, homozygous loss of ID2 results in profoundly disrupted rhythmicity, albeit with only partial penetrance, as well as in extensive reductions in wheel-running activity. This significant proportion of mice exhibiting disrupted and/or antiphasic diurnal rhythms is consistent with a role for *Id2* in either the SCN clock or output signaling from the SCN to the relevant relay centers in the central nervous system [7, 24–27]. A significant delay in the phase angle was also observed in the locomotor activity rhythms of *Id2* null mice. Because there is no significant difference in period lengths between genotypes, these data indicate that some *Id*2^−/−^ mice exhibit an abnormal phase relationship with the transition from lights on to lights off.

However, the most striking finding was that *Id2* null mice re-entrain to a large phase delay twice as fast as their littermate controls, as if ID2 acts as a brake on the circadian system in terms of its response to large changes in the light-dark cycle [28]. Interestingly, all four *Id* genes cycle in phase with one another within the SCN, whereas in other tissues, such as the heart, the phase relationships are more widely distributed over the circadian day. This phase consistency might underlie the rigid limitations of the SCN clock in its response to changes in the environment during the phase-delay portion of circadian cycle.

A photoentrainment phenotype similar to that of the *Id2* null mouse has recently been observed in *mPer1* mutant mice [29–31]. These mice show an enhanced phase delay to continuous treatments of light, e.g., 8 hr and 12 hr, but no differences in phase responses to short light pulses. This surprising coincidence suggests that ID2 and the canonical clock protein mPER1 may contribute to photoentrainment in similar ways. Also, similar to *Id2*^−/−^ mice and depending upon the study, *mPer1*-deficient mice can show normal circadian period lengths [32] and either normal or disturbed rhythms in DD [30, 32, 33].

Given that the ID proteins are known to interact with bHLH transcription factors, we tested the obvious hypothesis that the ID proteins might influence the activity of CLOCK and BMAL1. ID1, ID2, and ID3 were each shown capable of blocking CLOCK:BMAL1-driven transcriptional activation with a potency comparable to mPER1, suggesting a potential interplay between these transcriptional inhibitors and the bHLH transcriptional activators in the circadian feedback loop. The simplest interpretation is that ID proteins interact directly with CLOCK and/or BMAL1, reducing CLOCK:BMAL1 heterodimer binding to the E-box element and thereby muting the photic response at the level of clock genes (see Supplemental Discussion). In contrast to the other family members, ID4 did not interfere with CLOCK:BMAL1-driven transcriptional activation, and it correlates with the absence of the photoentrainment phenotype in the *Id4*^−/−^ mice.

Although we were initially surprised not to see a greater effect of loss of *Id2* on circadian period length, it should be noted that all four *Id* gene products display significant similarities in tissue distribution, amino acid sequence, and binding-partner affinities [1, 12, 13, 34], and all displayed rhythmic expression where they were expressed. Given these similarities, evidence of a more profound role for ID2 in the circadian system may have been obscured by functional redundancy with other members of the *Id* gene family [1], especially in the SCN, where all four genes cycle in phase.

A role for clock-controlled ID proteins, and specifically ID2, in a loop that closes outside the core oscillator but that affects aspects of circadian timing is reminiscent of the action of VIVID in *Neurospora* [35]. Expression of *vvd* is controlled by the clock, and VVD in turn feeds back to regulate the expression of several input and output genes yet is not required for circadian rhythmicity. Other examples of additional autoregulatory loops that contribute to the circadian system, but are not essential for the persistence of the oscillator, include *Dec1* and *Dec2*, *clockwork orange*, CREB, CREM, and the *frequency* antisense transcript [6, 36, 37].

## Supplemental Data

Document S1. Supplemental Results and Discussion, Supplemental Experimental Procedures, One Table, and Ten Figures
