# Supplementary material for: A Role for Id2 in Regulating Photic Entrainment of the Mammalian Circadian System
Source: Curr Biol. 2009 Feb 24;19(4):297–304. doi: 10.1016/j.cub.2008.12.052 (PMC2648875; doi:10.1016/j.cub.2008.12.052)
Supplement: Document S1. Supplemental Results and Discussion, Supplemental Experimental Procedures, One Table, and Ten Figures [file mmc1.pdf]

Current Biology, Volume *19*

Supplemental Data

A Role for Id2 in Regulating Photic Entrainment of the Mammalian Circadian System

Giles E. Duffield, Nathan P. Watson, Akio Mantani, Stuart N. Peirson, Maricela Robles-Murguia, Jennifer J. Loros, Mark A. Israel, and Jay C. Dunlap

Supplemental Results and Discussion

Id gene expression

An extensive examination of DNA microarray studies was taken to assess occurrence of *Id* genes as being identified as rhythmically expressed with a circadian period length [1-10]. *Id* genes were identified as rhythmic in over half of the studies examined: *Id1* in the pineal and immortalized 2.2 SCN cells, *Id2* in SCN, liver, aorta (via the Genomics Institute of the Novartis Research Foundation, GNF, 'Database of Gene Expression' http://expression.gnf.org/cgi-bin/circadian/index.cgi, developed by J. Hogenesch and A. Su) and rat-1 fibroblasts, and *Id3* in the SCN and immortalized 2.2 SCN cells [2, 4-7, 9]. The recent unpublished addition of a circadian time-course of mouse kidney to the GNF 'Database of Gene Expression', also shows *Id2* expression to be significantly rhythmic. Hughes et al (2007) [10] and associated web site http://wasabi.itmat.upenn.edu/circa/mouse/ (developed by the Panda and Hogenesch laboratories), allows for more precise determination of rhythm and phase. In this study, in which RNA was

analyzed at a 1 hr versus a 4 hr interval, it is possible to objectively assess rhythms with more confidence and precision. Use of this website has allowed us to determine, using the same statistical stringency of the COSOPT statistical determination of MMC-ß value ≤ 0.10 employed in Panda et al (2002) [2], that *Id2*, *Id1* and *Id4* show rhythmic profiles in the liver with a high level of significance. Note that a lack of concordance in genes identified between DNA microarray analyses of identical tissues, microarray platforms and comparable experimental conditions is common, which would explain these specific inter-study differences [8, 10, 11].

***Id2* null mouse and developmental considerations**

We designed a replacement vector to delete two exons that contain the entire coding region of *Id2*, full details of which can be found in the Supplemental Experimental Procedures. In brief, targeted deletion of the *Id2* transcript was confirmed by qRT-PCR from mRNA extracted from *Id2*+/+ and *Id2*-/- adult brain and liver, and mouse embryonic fibroblasts, and by protein analysis from adult liver (Figure S2). The 2.2 kb mutant protein product of Neo-Exon3 contains no known functional domains as Exon 3 is wholly 3' UTR and contains no coding sequence, and thus is unlikely to form any functional protein.

An independently generated *Id2* knockout mouse has been generated by Yokota and colleagues [12] and has revealed a functional role for ID2 in the development of the immune, mammary gland, heart and adiposite systems. The most overt phenotype is retarded growth [12]. In the case of the regulation of the immune system, specifically in the development of peripheral lympoid organs and generation of natural killer cells (absence of lymph nodes and Peyer's patches and impaired NK-cell production) [12], reduction in numbers of intestinal intraepithelial lymphocytes [13], altered T

cell and dentritic cell populations [14], and regulation of B cell IgE class switching [15]. A defect in lactation associated with mammary gland development during pregnancy is associated with epithelial cell proliferation arrest [16]. Cardiac structural and functional conduction system abnormalities have been documented that result in left bundle branch block [17]. A partial penetrance has been described for abnormal development of the ureteropelvic junction resulting in hydronephrosis [18]. A reduced adiposity, and embryonic fibroblasts derived from these mice exhibit a diminished capacity for adipocyte differentiation [19].

Whilst we have not explored any specific studies of the immune system in our *Id2* null mouse, we have observed considerable loss of animals during post-natal and early weaning phase of development. Loss of post-weaned mice is higher when mice are removed from a barrier facility, and we have found that supplementation with sterile food, and antibiotic treated water increases survivorship. These findings are consistent with a defect in the immune system of the *Id2* null mice. We have also been unable to breed successfully *Id2* homozygote female with *Id2* heterozygote male crosses, presumably due to the lactation defect, as identified in the Yokota *Id2* knockout mouse. Consistent with a low adiposity in the Yokota *Id2* knockout mouse, we find that the *Id2* null mice have a low level of fat storage and are lean (Hou, Watson, Israel and Duffield, unpublished data), contributing in part to the low body weight measurements.

Modulation of the photic response by ID2

Loss of ID2 has a clear effect on the photic response, and because the ID proteins can interact with and modify the activity of bHLH transcription factors, we reasoned that this influence might underlie the photic response phenotype. To test this we performed the experiments described in the

text showing that ID1, ID2 and ID3 were each shown capable of blocking CLOCK:BMAL1-driven transcriptional activation with a potency comparable to mPER1, with ID2 showed the greatest inhibitory effect. The potent ability for ID proteins to interfere with the CLOCK:BMAL1 activation of clock gene and clock-controlled gene activity reveals a potential interplay between the ID HLH transcriptional inhibitors and the CLOCK and BMAL1 bHLH transcriptional activators in the circadian transcriptional-translational feedback loop. This would provide a novel manner in which additional components, namely *Id* genes, could modulate circadian function. In the context of CLOCK and BMAL1 proteins, which function as a heterodimer bound to DNA, the simplest interpretation of our results posits that ID proteins are binding directly to CLOCK and/or BMAL1 and in turn reducing the quantity of functional CLOCK:BMAL1 heterodimers available for binding to the E-box element.

This suggests that under appropriate circumstances, reduction or absence of ID2 could result in higher activity of CLOCK:BMAL1, in turn facilitating a stronger response to photic stimuli via transactivation of the *mPer1*and *mPer2* gene promoters. As has been demonstrated, modulation of the quantity of functional CLOCK:BMAL1 heterodimer in the *Clock* mutant mouse results in changes in the induction profiles of the clock state variable genes, *mPer1* and *mPer2* [20, 21]. These studies suggest that CLOCK:BMAL1 activity, whilst not serving actually to mediate the photic signal activating the *period* genes (this being pCREB [22-24]), can still modify the magnitude of the response to the photic stimulus. Additionally, a direct role for CLOCK in signaling to the *period1* gene has also been proposed independent of the pCREB pathway: Ca2+ -dependent protein kinase C phosphorylation of CLOCK can regulate *period 1* induction [25].

Supplementary Table S1. Phenotypic Characteristics of *Id2* mutant Mice

**Parameter**		**Genotype**		**Statistics**	
	***Id2* +/+**	***Id2* +/-**	***Id2* -/-**	**ANOVA**	**p value**
**Freerunning period length in DD (hr)**	23.78 ± 0.10	23.62 ± 0.05	23.89 ± 0.10	$F_{2,39}$ = 2.2	p = 0.1202, n.s.
**Duration of 'nocturnal' activity (*alpha*) (hr) 1**					
LD:	11.34 ± 0.61	10.08 ± 0.45	11.30 ± 0.62	$F_{2,35}$ = 1.5	p = 0.235, n.s.
DD:	12.18 ± 0.45	11.47 ± 0.67	12.09 ± 0.28	$F_{2,32}$ = 0.61	p = 0.5498, n.s.
**Activity level (wheel counts)**					
LD:	23674 ± 1278	23846 ± 3045	7687 ± 2154*	$F_{2,39}$ = 18.0	p = 0.0001*
DD:	31643 ± 2577	24309 ± 3440	10640 ± 3093*	$F_{2,37}$ = 12.9	p = 0.0001*
**Activity level (passive infrared detector counts)**					
LD:	2878 ± 431	3100 ± 796	3358 ± 374	$F_{2,24}$ = 0.26	p = 0.7752, n.s.
DD:	2425 ± 529	2745 ± 305	3120 ± 306	$F_{2,26}$ = 0.70	p = 0.5097, n.s.
**Fourier analysis *power* measure (circadian amplitude)**					
wheel, LD:	0.249 ± 0.026	0.273 ± 0.035	0.155 ± 0.03*	$F_{2,39}$ = 4.5	p = 0.0179*
wheel, DD:	0.241 ± 0.029	0.214 ± 0.025	0.137 ± 0.031*	$F_{2,35}$ = 3.7	p = 0.037*
passive, LD:	0.057 ± 0.019	0.171 ± 0.05	0.135 ± 0028	$F_{2,26}$ = 1.58	p = 0.2302, n.s.
passive, DD:	0.097 ± 0.054	0.156 ± 0.032	0.103 ± 0.024	$F_{2,26}$ = 0.40	p = 0.6783, n.s.
**Proportion activity during day/subjective day (ZT/CT12-ZTCT0) (%)**					
wheel, LD:	8.8 ± 1.9	3.6 ± 1.1	11.2 ± 2.4	$F_{2,39}$ = 2.4	p = 0.0631, n.s.
wheel, DD:	2.3 ± 0.7	2.6 ± 1.3	4.8 ± 1.9	$F_{2,35}$ = 0.94	p = 0.4003, n.s.
passive, DD **2**	14.5 ± 2.4	16.0 ± 3.6	16.3 ± 2.3 (47.7 ± 1.7*)	$F_{3,25}$ = 22.8	p = 0.0001*
**Time of peak activity relative to ZT12 or CT12 (hr)**					
LD:	2.69 ± 0.53	3.84 ± 0.64	4.26 ± 0.79	$F_{2,39}$ = 1.78	p = 0.1827, n.s.
DD:	3.96 ± 0.46	3.23 ± 0.59	4.92 ± 0.88	$F_{2,35}$ = 1.58	p = 0.2225, n.s.
**Phase of activity onset in LD**					
phase angle relative to lights-off (ZT12) (min) **3**	24 ± 11	40 ± 21	-3 ± 7*	$F_{2,37}$ = 2.93	p = 0.0661
variance of phase angle	1504	5299	680		
**No. days for stable entrainment following 10 hr delay of photoperiod (days)**	3.75 ± 0.19	3.11 ± 0.39	1.46 ± 0.26*	$F_{2,27}$ = 18.8	p = 0.0001*
**Phase shift from 10 hr change in photoperiod (hr)**	3.54 ± 0.60	3.59 ± 0.45	5.87 ± 0.57*	$F_{2,31}$ = 5.8	p = 0.0071*
**Phase shift from 30 min light pulse (min) at CT14**	121 ± 13	150 ± 7	126 ± 10	$F_{2,32}$ = 2.24	p = 0.1087, n.s.
**Phase shift from 30 min light pulse (min) at CT16**	98 ± 6	n/a	86 ± 15	$t_{1,26}$ = 0.70	p = 0.4876, n.s.
**Freerunning period length in LL (80 lux) (hr)**	25.28 ± 0.13	25.51 ± 0.23	25.48 ± 0.28	$F_{2,14}$ = 0.29	p = 0.7558, n.s.
**Proportion mice exhibiting arrhytmia in LL (80 lux) (%)**	38	33	40		

[1]Two-factor ANOVA reveals an effect of LD versus DD on *alpha* ($F_{1,67}$ = 5.2, p = 0.0257*); [2]One-factor ANOVA performed on four groups, with a separate group for the *Id2*-/- mice exhibiting disrupted rhythms; [3]Student's t-test (two-tailed): *Id2*+/+ versus *Id2*-/-, $t_{1,25}$ = 2.3, p = 0.0296*

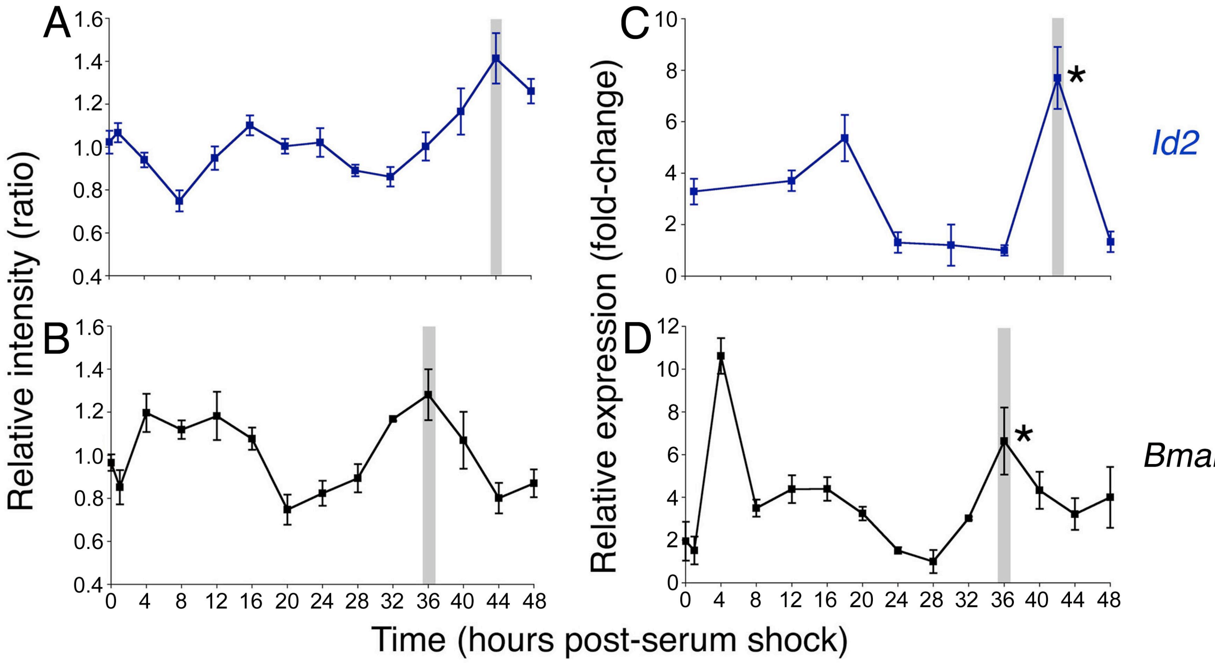

Figure S1. ***Id2*** **is rhythmically expressed in rat-1 and mouse NIH3T3 fibroblasts**

Gene expression analysis of serum-treated confluent fibroblasts (2 hr serum treatment started at time = 0 hr).

(A, B) cDNA microarray analysis of rat-1 cells, showing rhythmic profiles of *Id2* (A, top left) and *Bmal1* (B, bottom left) Values are mean ± SEM ratio of time-specific expression relative to medial expression values from five independent experiments (n = 3-5 each time point).

(C, D) Real-time quantitative RT-PCR of mouse NIH3T3 cells, showing rhythmic profiles of *Id2* (C, top right) and *Bmal1* (D, bottom right). Values are mean ± SEM ratio of expression relative to the lowest expression value from three independent experiments (*Id2* and *GAPDH* using *Taqman* reagents, and *Bmal1* using SYBR green, ABI 7700, normalized to *GAPDH*).

Peak phase of rhythms are indicated by a grey bar. Statistical analysis of microarray and qRT-PCR samples reveals significant rhythms (DNA microarray data, CORRCOS, *p < 0.05 [4]; qRT-PCR data, one-factor ANOVA, followed by Dunnett's post-hoc t-tests, *p < 0.05).

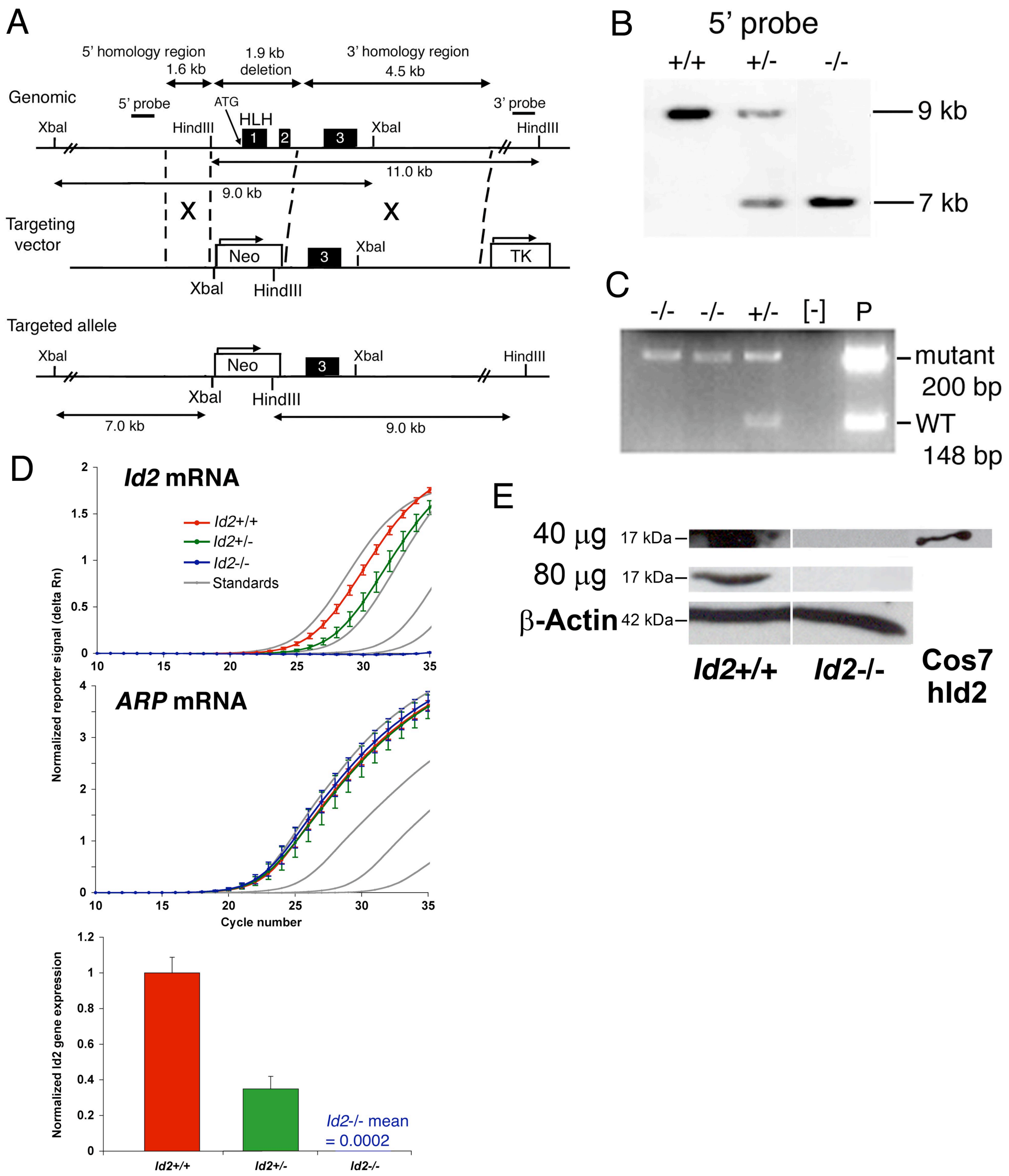

Figure S2. Generation of *Id2-/-* mutant mice

(A) Schematic representation of the *Id2* gene, the recombinant targeting construct, and the resulting targeted allele. A 1.9 kb portion of the *Id2* gene, including 560 bp 5' upstream of the transcription

start site (containing a significant proportion of the promoter region) and exons 1 and 2, was replaced by a 1.8 kb neomycin resistance cassette (Neo). Boxes represent exons, labeled 1-3, and the lines between the exons represent introns. Horizontal filled boxes indicate the locations of probes used for Southern blotting. Dashed lines and crosses indicate the 5' and 3' regions of homology used for homologous recombination. Solid lined arrows indicate fragment sizes detected by 5' and 3' probes following *Xba*I and *Hind*III digest of genomic DNA, respectively. Neo, neomycin resistance gene; TK, Herpes simplex virus *thymidine kinase* gene; HLH, the helix-loop-helix domain encoded by exon 1.

(B) Southern blot analysis identification of the targeting event in mouse tail biopsies using a probe complementary to a 5' homology region. Representative autoradiogram showing bands of 9 kb and 11 kb, indicating the presence of the wild-type and mutant alleles, respectively. Genotypes are shown above lanes. A probe complementary to the 3' homology region was used in Southern analysis to confirm correct targeting.

(C) PCR analysis of genomic DNA extracted from mouse tails. Genotypes are shown above lanes. [-], distilled water; P, full length *Id2* cDNA and neomycin resistance gene plasmid plasmid as a positive control.

(D) Quantitative real-time RT-PCR analysis of mRNA extracted from F2 adult mouse liver using primers specific for 5' region of the *Id2* cDNA show no measurable quantity of *Id2* transcript in samples derived from *Id2*-/- mice. Mean ± SEM normalized reporter signal (delta Rn) versus cycle number shown for *Id2*+/+ (red, n = 11 mice), *Id2*+/- (green, n = 3 mice) and *Id2*-/- (blue, n = 5 mice). *Id2* gene expression using *Taqman* reagents was normalized to acidic ribosomal phosphoprotein (ARP) using SYBR green, ABI 7700. Standards are 100, 10, 1 and 0.1 ng total RNA. Values in histogram are mean ± SEM ratio of expression relative to the mean *Id2*+/+

expression value = 1.0. qRT PCR of F2 adult mouse brain and mouse embryonic fibroblasts showed consistent results (data not shown).

(E) Western blot analysis of protein from F2 adult mouse liver shows no measurable quantity of ID2 protein in *Id2*-/- mice. Representative blots are shown using 40 μg and 80 μg of total protein from *Id2*+/+ and *Id2*-/- liver, and from Cos7 cells transfected with a human Id2 expression plasmid.

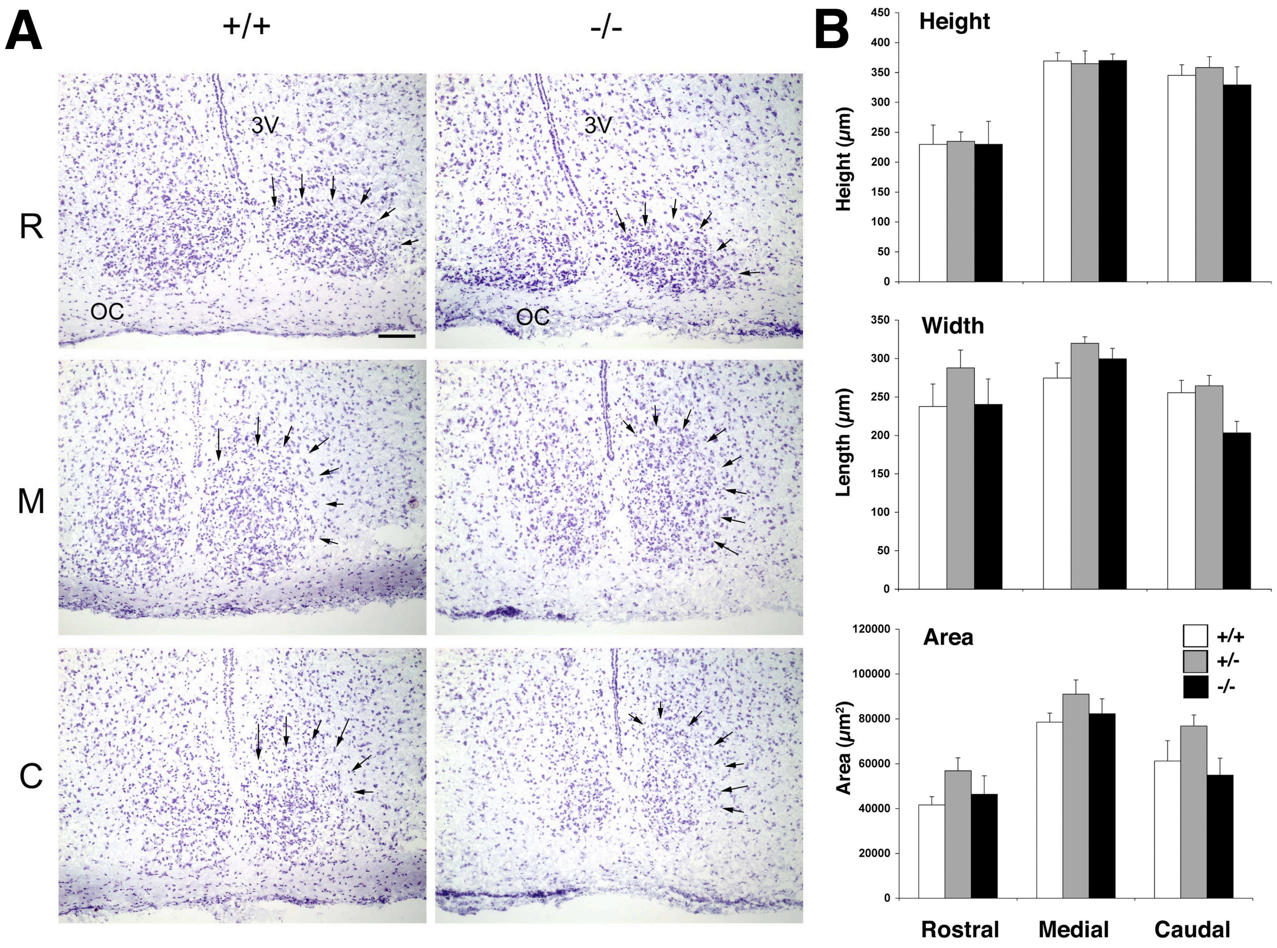

Figure S3. Histological analysis of suprachiasmatic nucleus shows no gross anatomical difference between *Id*2+/+ and *Id2*-/- F2 littermates

(A) Cresyl violet stained coronal sections through rostral (R), medial (M) and causal (C) aspects of *Id2*+/+ and *Id2*-/- SCN. Arrows indicate perimeter of SCN. OC, optic chiasm; 3V, third ventricle. Scale bar = 100 μm.

(B) Analysis of height, width and area of rostral, medial and causal aspects of SCN. Values shown in the histogram are group means ± SEM for wild-type (white), heterozygote (grey) and *Id2*-/- (black) mice. No significant differences were detected between genotypes (one-factor ANOVA for each measurement x each aspect, with FDR Correction).

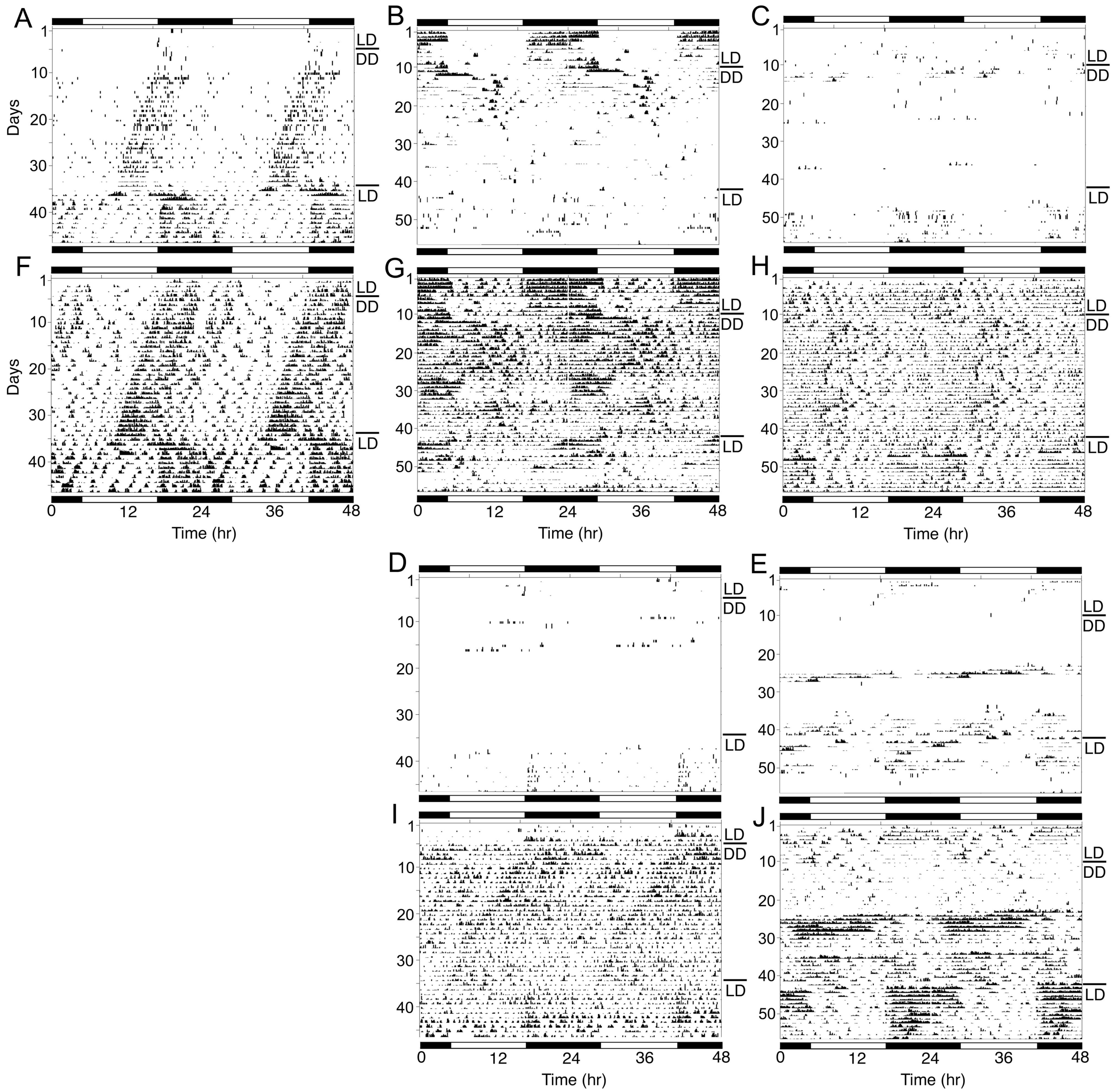

Figure S4. Locomotor activity traces of *Id2* mutant mice with disrupted circadian rhythms

(A-J) Wheel running and corresponding passive activity records of *Id2* mutant mice are shown in double-plotted format. Each horizontal line represents a 48 hr period and the second 24 hr period is plotted to the right and below the first. Vertical bars represent periods of wheel running activity (A-E) or corresponding general activity as measured by a passive infrared detector (F-J). Animals were

individually housed in a 12 light:12 dark cycle (LD) for at least 14 days, transferred to constant darkness for 30 days (DD), and then transferred back to a LD cycle. The line above DD on the right indicates the transition from LD to DD. The timing of the respective light-dark cycles are indicated by the white/black bars above and below the records. Numbers on the left indicate the number of days in the study. Of the *Id2* mutant mice, 12 mice showed distinct to robust rhythms in both LD and DD, as shown in the representative animal (A, F) and in **Figure 2C**, whilst 4 mice became arrhythmic or showed very weak rhythmicity in DD (B-E; G-J). Note the example mouse in (A) and (F) was specifically presented as it showed a low amount of wheel running activity, but dissimilar to (B)-(E) and (G)-(J), and despite this behavior, it demonstrated a robust circadian rhythm in DD. Note also that mice represented in (B-C, E) also received a 10 hr treatment of light starting at ZT12 on the last day on LD (day 10), and in which a phase delay of the rhythm can be observed. These mutant mice showed severe disruption of their wheel running activity in DD, but did show some measure of activity when maintained on a LD cycle (B-E). Despite the reduction or absence of activity within the available running-wheel, the *Id2* mutant mice remain active. The arrhythmic or very weak rhythmicity cannot be explained by a reduced preference to run in the available wheel. In fact, wild-type and heterozygous mice examined in the absence of a running wheel, still demonstrate robust rhythms of passive activity (**Figure 2B**). The corresponding quantity of passive activity was not noticeably reduced in the phenotypic *Id2* mutants when in DD compared to in LD (paired t-test, n.s.).

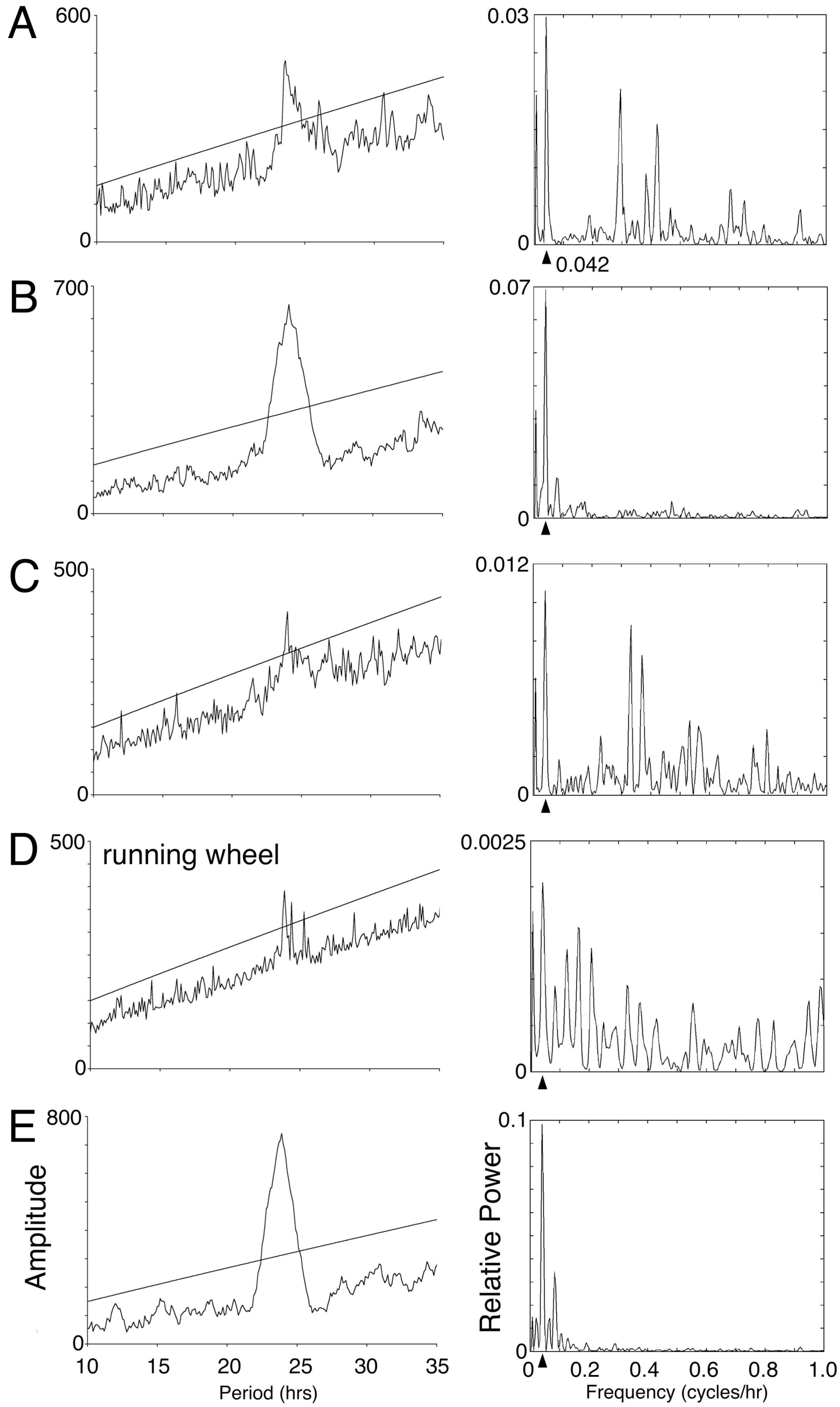

Figure S5.

χ^2 Periodogram and Fourier analysis of the locomotor activity rhythms of *Id2* mutant mice in LD

(A-E) Plots represent analysis of 10 days of data in LD from a representative *Id2* mutant mouse with a normal circadian behavior pattern in DD (A), and four *Id2* mutant mice with disrupted rhythms in DD (B-E). Analyses correspond to those mice whose actograms are shown in **Figure S4 (A-E and F-J)**. The analyses were undertaken on the last 10 day duration in LD. Analyses are of passive activity data, except in (D) where the mouse showed significant rhythmicity in LD only on analysis of the wheel-running activity data. For the

periodogram analysis, the ascending straight line represents a statistical significance of p = 0.001. For Fourier analysis, a frequency of 0.042 cycles/hr corresponds to 1 cycle/24 hr, which is denoted by the arrow head below the chart where statistically significant (statistical significance was determined by the *Clocklab* program). All five mice in LD showed significant but in some cases extremely weak rhythmicity when measured from either passive or locomotor activity data.

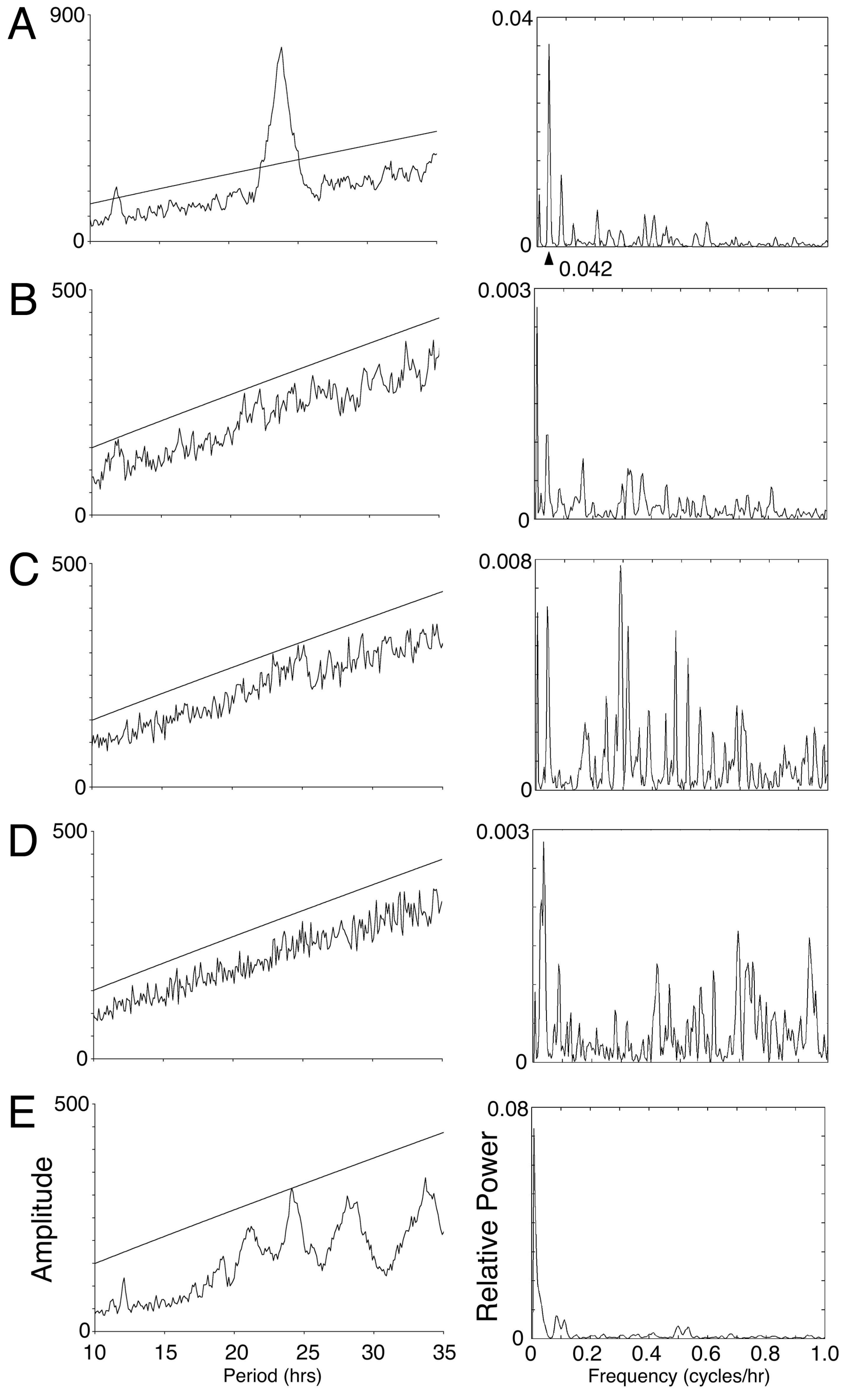

Figure S6.

χ^2 Periodogram and Fourier analysis of the locomotor activity rhythms of *Id2* mutant mice in DD

(A-E) Plots represent analysis of 10 days of data from a representative *Id2* mutant mouse with a normal circadian behavior pattern in DD (A), and *Id2* mutant mice with disrupted rhythms in DD (B-E). Analyses correspond to those mice whose actograms are shown in **Figure S4 (A-E and F-J)** and whose LD periodograms and Fourier analyses are shown in **Figure S5**. The analyses were undertaken on the third 10 day duration in DD. Analyses are of passive activity data only. For the periodogram analysis, the ascending straight line represents a

statistical significance of p = 0.001. For Fourier analysis, a frequency of 0.042 cycles/hr corresponds to 1 cycle/24 hr, which is denoted by the arrow head below the chart where statistically significant (statistical significance was determined by the *Clocklab* program). Note the changes in the y-axis between charts between this figure and **Figure S5**, and a reduction in values indicates a loss of rhythm amplitude in DD. In DD, no significant circadian rhythms were detected from either passive activity or wheel-running activity (periodogram and Fourier analyses not shown) in these four particular *Id2* mutant mice. Analysis of the *Id2* mutant mouse in (A) showed significant rhythms in both LD and DD.

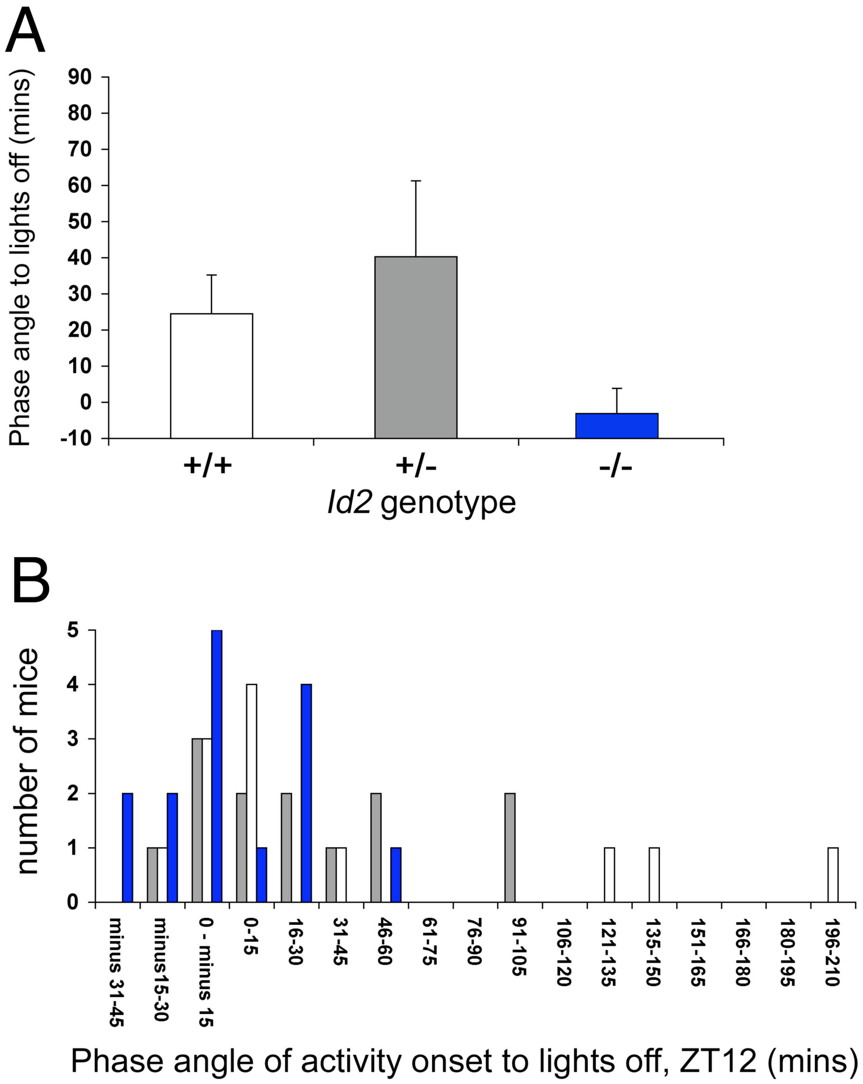

Figure S7. *Id2* mutant mice exhibit a delayed phase angle of activity onset relative to lights off (ZT12)

(A) Phase-angle of activity onset to lights OFF (Zeitgeber time 12, ZT12) of 12:12 LD cycle. Values shown in the histogram are group means ± SEM for wild-type (white), heterozygote (grey) and *Id2*-/- (blue) mice. The values marked by asterisk are statistically significant (one-factor ANOVA, $F_{2,37} = 2.93$, $p = 0.066$; two-tailed t-test for *Id2*+/+ vs. *Id2*-/-, $t_{1,25} = 2.3$, $*p < 0.05$).

(B) Histogram of the distribution of the phase-angle of activity onset determined for wild-type (white), heterozygote (grey) and *Id2*-/- (blue) mice on 12:12 LD cycle.

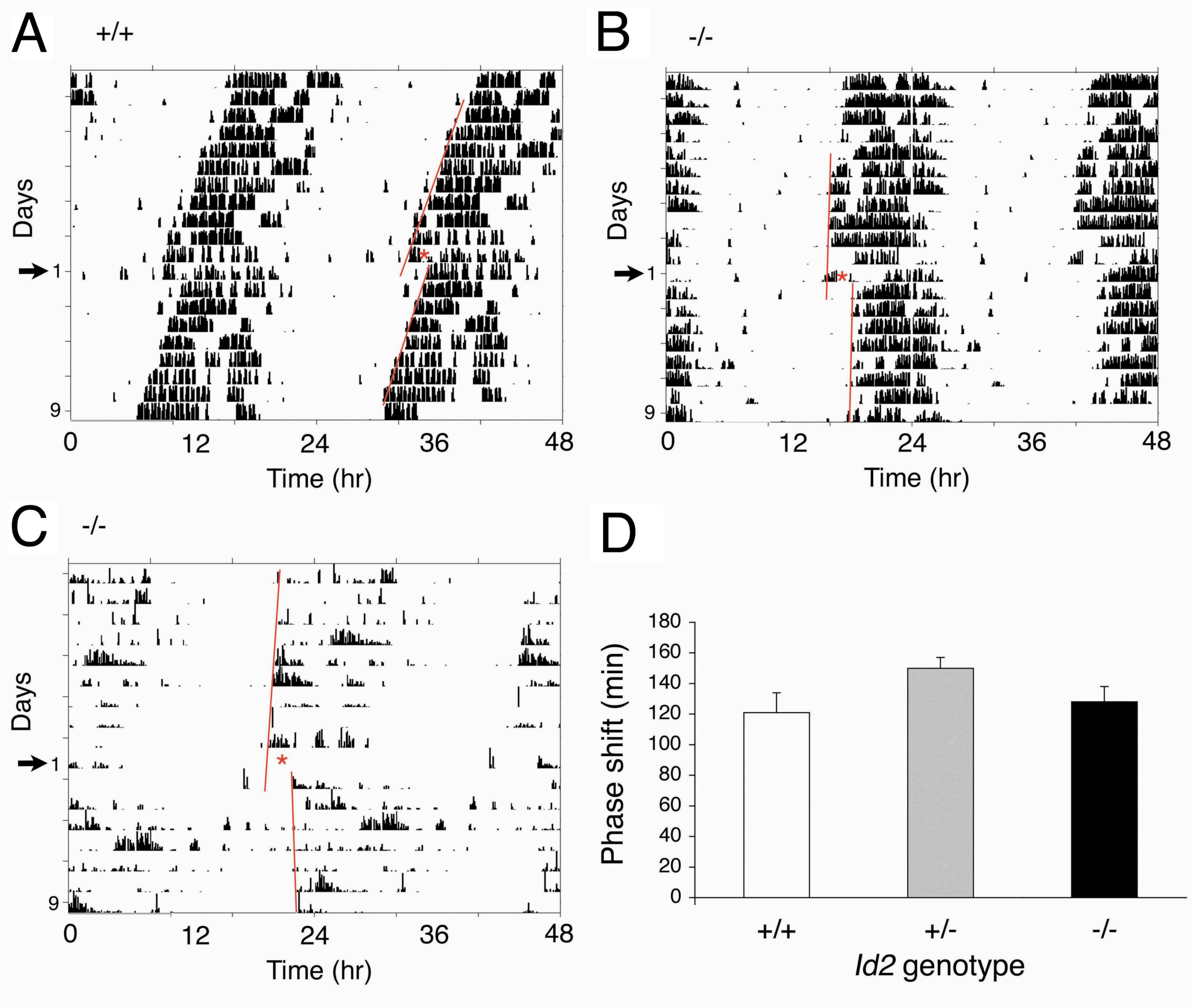

Figure S8. There is no effect of genotype upon the magnitude of non-parametric light-induced phase shifts

(A-D) Activity records of representative wild-type (+/+; A), and *Id2* mutant (-/-; B, C) mice in DD shown in double-plotted format, and histogram (D) summarizing the mean ± SEM light-induced phase shifts (n = 10, 13, 12 for wild-type, heterozygote and mutant mice, respectively). Animals were individually housed in a 12 light:12 dark cycle (LD) for at least 14 days and transferred to constant darkness (DD) 9-11 days prior to treatment with a 30 min light pulse delivered at CT14. Numbers on the left indicate the number of days following the light treatment, with day 1, marked

by the arrow on the left, being the day of treatment. The time of light pulse is also indicated by an asterisk within the actogram. A line is fitted to the phase of activity onset before and after the light treatment, and the time difference between the two lines is the measured phase delay or phase advance of the free-running rhythm. No significant difference was detected between genotypes (one-factor ANOVA, *$p < 0.05$).

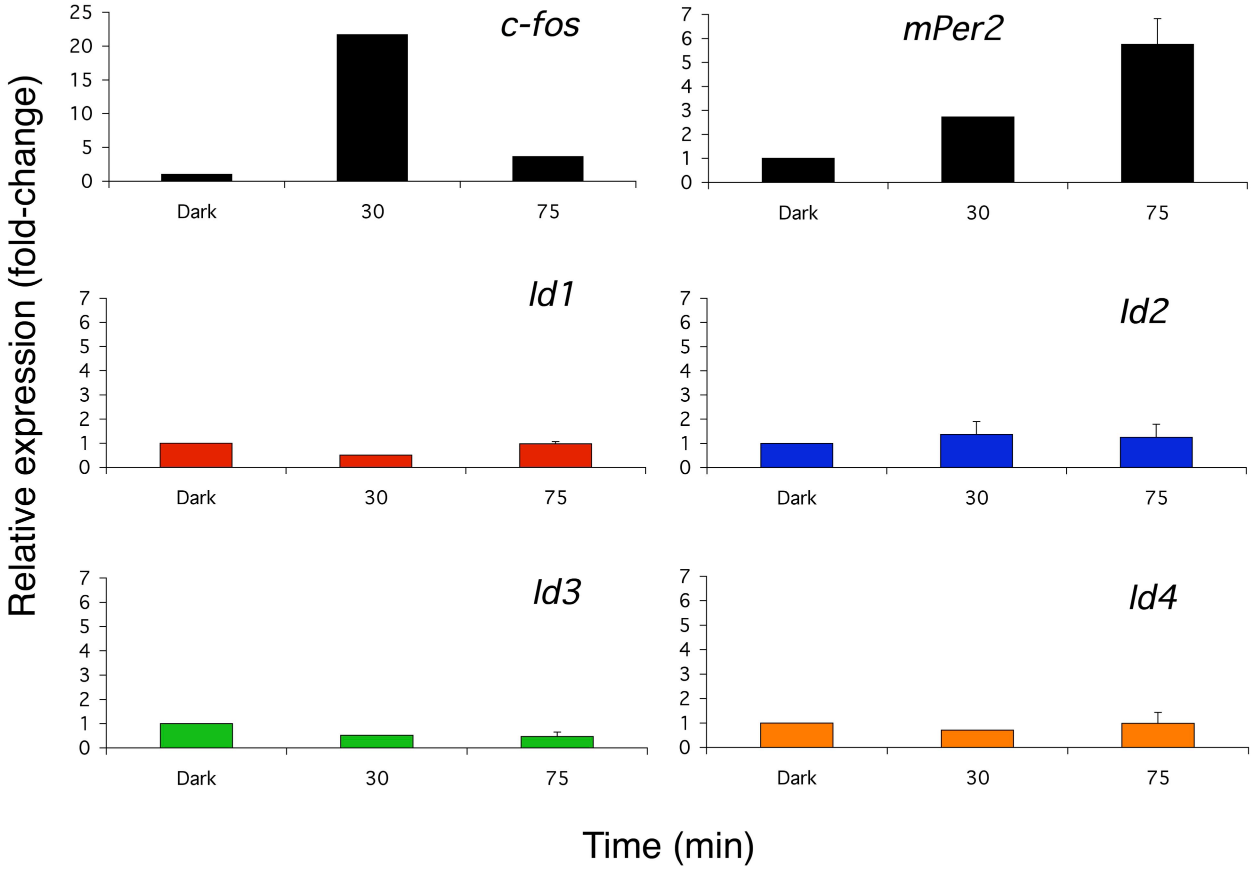

Figure S9. *Id* mRNA is not induced in the SCN following light pulse treatment

Mice (C3H/He) were exposed to a 30 min light pulse (800 lux) at CT16, and SCN collected at 30 min and 75 min after start of treatment. Mice were maintained for 1-3 circadian cycles in DD following transfer from a 12:12 LD cycle. Tissue from dark pulse control mice collected at CT16.5-17.5. Gene expression analyzed by quantitative real-time qRT-PCR for the genes *c-fos, mPer2* and *Id1-4* (SYBR green, ABI 7700, normalized to *18S* and *ARP*). *c-fos* and *mPer2* are induced by light in a predictable manner at 30 min and 75 min, respectively. *Id* genes showed no major change (> 2 fold relative to dark pulsed controls) at either time point following light treatment. Data are mean ± SD pooled SCN samples from two independent experiments.

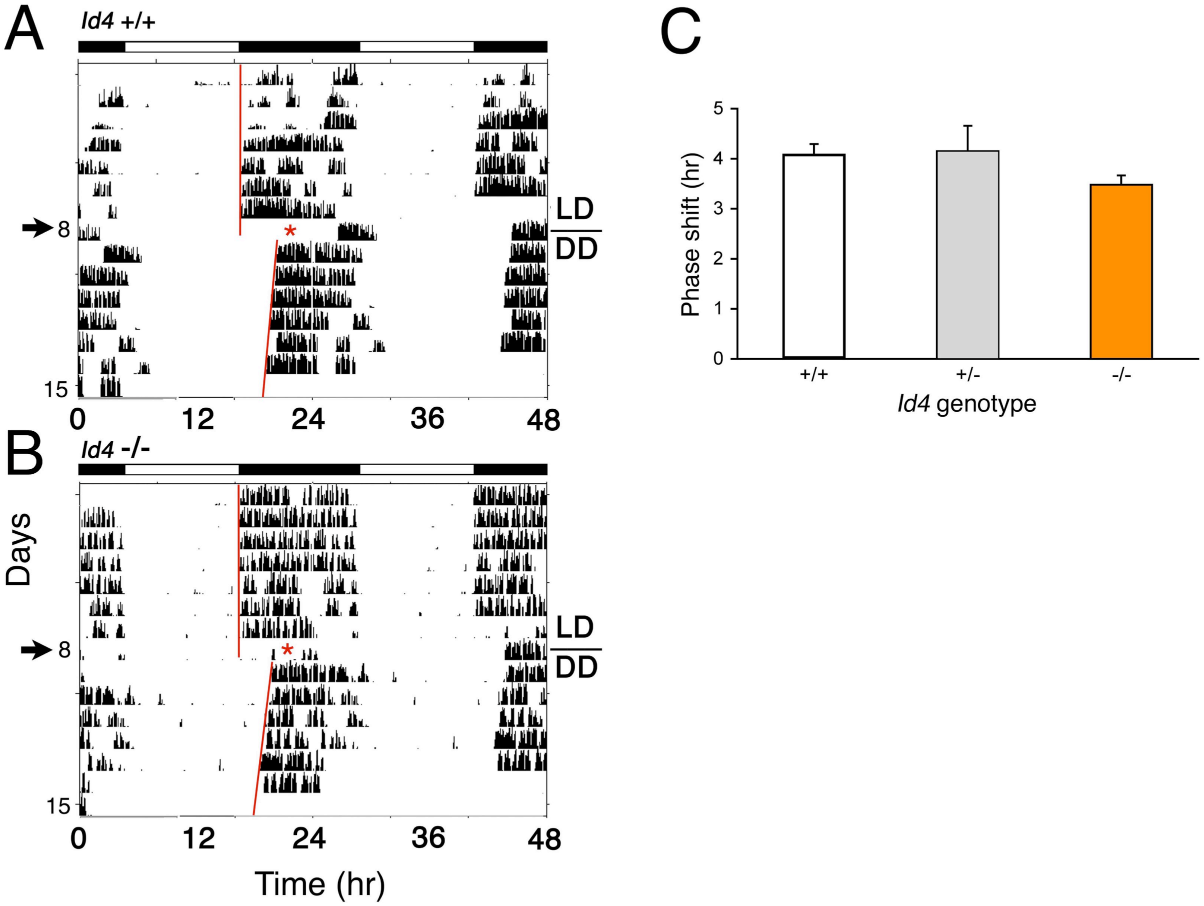

Figure S10. *Id4* mutant mice show normal entrainment response to an extension of the light phase in the daily photoschedule

(A, B) Representative locomotor activity records of wild-type (A) and *Id4* mutant (B) exposed to a 10 hr extension of the light phase of the LD cycle. Mice were maintained on a LD cycle for at least 14 days and transferred to a new LD cycle where ZT12 is delayed by 10 hr by extending the light phase of the LD cycle by 10 hr on day 1, and then transferred to DD for the rest of the experiment. The line above DD on the right indicates the transition from LD to DD. The timing of the respective light-dark cycle is indicated by the white/black bars above the records. Numbers on the left indicate the number of days following the transition to the new LD cycle or the 10 hr light treatment prior to transfer to DD. The arrow on the left indicates the actual day of treatment (day 1), and the actual

mid-time of treatment is marked by an asterisk within the actogram. A line is fitted to the phase of activity onset for several days before and after the transfer to DD.

(C) The mean ± SEM magnitude of the phase shifts produced by light treatment is shown in the histogram for wild-type (white, n = 10), heterozygote (grey, n = 9) and mutant (orange, n = 10) mice. Extrapolated activity onsets of the first day following the 10 hr light treatment were used to determine the size of resultant phase delays. No significant difference was detected between genotypes (one-factor ANOVA, *$p < 0.05$).

Supplemental Experimental Procedures

Cell treatment and RNA collection

Confluent Rat-1 and mouse NIH3T3 fibroblasts (ATCC, Manassas, VA) were treated with 50% horse serum, RNA harvested, and circadian patterns of gene expression analyzed by cDNA microarray and qRT-PCR, as detailed previously [4] and below, respectively. The start of serum treatment was designated time = 0 hr and gene expression was examined every 4-6 hr over a 48 hr period.

Mouse tissue collection

Mice (C3H/He wild-type [26]; n = 6 per time point) were housed under a 12:12 LD cycle (1000 lux, fluorescent lights, General Electric 70-W cool white: 0 lux) with food and water *ad libitum* for 14 days before being transferred to constant dark conditions (DD) for 3 days. Animals were sacrificed by cervical dislocation at 4 hr intervals, at circadian time (CT) 0, 4, 8, 12, 16 and 20, according to Schedule 1 of the UK/British Home Office Animals (Scientific Research) Act, and as approved by

the Institutional Animal Care and Use Committee at Dartmouth Medical School. For the light pulse experiment, animals were sacrificed at 30 min and 75 min following the start of a 30 min (800 lux) light treatment initiated at CT16, or dark pulse controls collected between CT16.5 and CT17.5. The intervals between start of light stimulus and tissue collection was based on the profile of *Id2* induction following mitogenic serum stimulation of fibroblasts [27], and the profiles of immediate early gene (e.g. *c-fos*) and clock gene (*mPer1* and *mPer2*) induction in the SCN [28-31]. Brains were rapidly removed and snap frozen in isopentane at –60 °C on dry ice for 20 s before a 1mm coronal section was cut at the level of the optic chiasm using a fabricated twin blade cutter. A SCN punch from a frozen slice was then taken under a stereomicroscope using a flat-tipped 25G needle (internal diameter approximately 0.5 mm) and tissue stored on dry ice. The shape of the optic chiasm and third ventricle were used to define the SCN region. All six SCN punches were pooled to obtain sufficient RNA for subsequent procedures. The apical pole of the heart was dissected out and immediately placed into 0.5ml *RNA later* (Ambion, Austin, TX) and stored at 4 °C prior to RNA extraction.

Quantitative real-time RT-PCR analysis

Total RNA was prepared with Trizol reagent (Sigma-Aldrich, UK), DNAseI treated and cDNA synthesized as described previously [4, 32]. *SCN*: one pooled sample at all time points; *heart*: 3 samples at each time point. Quantitative Real-time RT-PCR (qRT-PCR) was conducted using SYBR green and *Taqman* reagents as described previously [4, 32]. Primers were designed for the clock genes *mPer2* (forward 5’-GGGGTGAGATTCGTCATTGAACTTG-3’, Reverse 5’-AGGACATTGGCACACTGGAAAGAG-3’) and *Bmal1*, (forward 5’-GAGGTGCCACCAACCCATAC-3’, reverse 5’- AGTCAAACAAGCTCTGGCCAA-3’), *c-fos* (as

described previously [33]), and the *Id* genes, *Id1* (forward 5'-GACGAGCAGCAGGTGAACG-3', reverse 5'-TTGCTCACTTTGCGGTTCTG-3'), *Id2* (forward 5'-AAGACTTTTGTTATCAGCCATTT-3', reverse 5'-GACGATCATCCTTAGTTTTCCTTCCGCTTTCTT-3'; Taqman primer and probe set, forward 5'-CCGCTGACCACCCTGAAC-3', reverse 5'-CATTCGACATAAGCTCAGAAGGGAATT-3', probe 5'-FAM- CGGACATCAGCATCCTGTCCTTGCAGGC-TAMRA-3', *Id3* (forward 5'-AGCCTCTTAGCCTCTTGGACG-3', reverse 5'-CACGCTGCAGGATTTCCAC-3') and *Id4* (5'-CTGTGCCTGCAGTGCGATAT-3', reverse 5'-CGTGCTGCAGGATCTCCAC-3'), and internal controls, 18S ribosomal RNA (18S rRNA; forward 5'-TTGTTGGTTTTCGGAACTGAGGC-3', reverse 5'- GGCAAATGCTTTCGCTCTGGTC-3'), *acidic ribosomal phosphoprotein* (*ARP*; forward 5'-CGACCTGGAAGTCCAACTAC-3', reverse 5'- ATCTGCTGCATCTGCTTG), and *GAPDH* primer and probe set (*Taqman Rodent GAPDH control reagents*, Applied Biosystems, Foster City, CA). Primers were designed using MacVector 7.0 software (Accelrys, UK) and synthesized by Sigma-Genosys (UK). All products were sequenced to confirm specificity of amplification. Real-time PCR was conducted using SYBR Green I Mastermix or Taqman Mastermix using an ABI PRISM™ 7700 Sequence Detection System (Applied Biosystems). Each reaction was run in triplicate and contained 1 μl of cDNA (25 ng total RNA) template along with 300 nM of primers in a final reaction volume of 25 μl. Cycling parameters were 95 °C for 10 minutes to activate the DNA polymerase, 40 cycles of 95 °C for 15 seconds, 60 °C for 1 minute (recording step), and for SYBR green reagent, with an additional recording step of 78-84 °C for 20 s to prevent any primer-dimer formation (temperature dependent upon the predicted melting temperatures of the specific PCR product; *mPer2*, 78 °C; *Bmal1*, 78 °C; *c-fos*, 78 °C; *Id1*, 81 °C; *Id2*, 77 °C; *Id3*, 81 °C; *Id4*, 81 °C; *ARP*, 76 °C; 18S rRNA, 78°c). Melting curves were performed

using Dissociation Curves 1.0 (Applied Biosystems) to ensure only a single product was amplified, and samples were also run on a 3% agarose gel to confirm specificity by size of band. Expression data from mouse SCN and heart RNA were normalized to the expression of two internal controls, 18S rRNA and *ARP*. Expression data from mouse NIH3T3 cells and mouse embryonic fibroblasts was normalized to the expression of *GAPDH*. The *Id2 Taqman* primer and probe set was used with RNA samples from NIH3T3 cells, and from adult brain and mouse embryonic fibroblasts of *Id2*+/+ and *Id2*-/- mice. Data were initially analyzed using SDS 1.7 (Applied Biosystems). Relative mRNA abundance was calculated using two methods: raw data were exported as clipped files and analyzed as efficiency-corrected normalized expression as described previously [33], and as no statistical differences in amplification efficiency were observed, the mean efficiency was used for each transcript; and using the standard curve method outlined in the *ABI Prism 7700 Sequence Detection System User Bulletin #2* (Perkin Elmer/Applied Biosystems) [4]. Significant differences were determined by one-factor ANOVA, followed by post-hoc Dunnett's t-tests ($p < 0.05$).

Generation of *Id2* mutant mice

A replacement vector was designed that deletes two exons that include the entire coding region of mouse ID2 protein. The targeted allele was obtained in embryonic stem cells (**Figure S2B**) and used to generate germline chimaeric mice. Intercrosses between heterozygous (C57BL/6 x 129) F1 offspring produced homozygous mutants at the expected Mendelian rate when examined at the embryo stage. However, in our recent studies, we observed a high level of lethality during the postnatal period and post-weaning, and thus we outbred the *Id2*-/- mice to increase litter size and reduce loss (see below). RT-PCR and sequence analysis indicated the presence of a mutant transcript that, if translated, would generate a protein with a deletion of the entire ID2 protein

product. Homozygous mutants are morphologically dissimilar to their wild-type littermate controls, being on average 27% smaller ($F_{2,56} = 9.1$, $p = 0.0004$), and our other broad observations of the *Id2*-/- mice are consistent with the *Id2* null mice generated by Yokota and colleagues [12]. Histological analysis of SCN coronal sections showed no gross anatomical difference between *Id*2+/+ and *Id2*-/- F2 littermates (**Figure S3**), indicating that the circadian phenotypes in the mutants are not due to a gross developmental defect in the basic organization of the SCN.

We isolated a genomic clone from a mouse 129/sv genomic P1 phage library (Genomic Systems, USA) using a 1.3 kb full length *Id2* complementary DNA probe [34]. A targeting vector was constructed with *Neomycin resistance* gene (*Neo*) as the positive selection marker and Herpes simplex virus *thymidine kinase* gene (*HSVtk*) as the negative selection marker to delete a 1.9 kb fragment. We used a *BamH*I-*Hind*III 1.6 kb fragment as the 5' homology region and a 4.5 kb *BamH*I-*BamH*I fragment as the 3' homology region. The final targeting construct was assembled by ligation of the *HSVtk* and *Neo* and vector backbone in the TV1 plasmid containing *Id2* genomic sequence.

In the targeting vector, the *Neo* cassette was inserted in forward orientation to the *Id2* allele, replacing 1.9 kb of the *Id2* gene including exons 1 and 2 (including the HLH domain) and 560 bp 5' upstream of the transcription start site and containing a significant proportion of the promoter region [34]. The targeting construct was linearized with *Not*I and introduced into JM-1 embryonic stem (ES) cells (129/svJ) by electroporation. Genomic DNA was extracted from neomycin-resistant ES cell lines, digested with *Xba*l or *Hin*III, and subjected to gel electrophoresis for Southern blot analysis.

Of the 200 resistant clones examined, 2 were found to have homologous recombination by Southern blot analysis using a probe from the 5'-flanking region and by PCR of genomic DNA.

Positive integration was identified by a 7.0 kb band corresponding to the mutant allele. A 9.0 kb fragment was produced in the wild-type background. No additional integration of the targeting construct was identified in these clones with a neo probe. A Southern blot analysis using a probe from the 3'-flanking region was used to confirm candidate cell lines, finding a 9.0 kb band corresponding to the mutant allele (data not shown). An 11.0 kb fragment was produced in the wild-type background. A positive ES cell line (no. 1-53) was microinjected to generate chimeric founders with germline transmission of the targeted allele.

Chimeric males were bred to C57BL/6J females, and F1 mice interbred to produce F2 mice. Homozygous mice were produced through heterozygous intercrosses. *Id2*-deficient mice were initially established on a C57BL/6J background, but for breeding purposes due to extremely low production of *Id2*-/- homozygotes (< 1% survivorship from heterozygote x heterozygote crosses), transgenic mice were later established in a mixed genetic background (129sv/C57BL6J/FBVN; resulting in ~10% survivorship from heterozygote x heterozygote crosses). Homozygote wild-type and heterozygote littermates were used as control mice in all subsequent experiments, as recommended [35].

Genotypes were determined by Southern blot analysis and/or PCR of tail biopsy DNA (**Figure S2**). Southern blot analysis was performed as described above. PCR primers specific for the wild-type and targeted alleles (neo) that were used (*Id2*-wild-type intron forward 5'-AGG CGC CAG TCT GCT TCT TGT AAC-3'; *Id2*-wild-type reverse in exon 3, 5'-CAA AAC TGT AGC CCT CTG AG-3'; neo forward 5'-TAG CCT GAA GAA CGA GAT CAG CAG-3'). PCR conditions (95 °C for 30 s, 56 °C for 40 s, 72 °C for 1 min, 30 cycles in 1x PCR buffer [Promega] containg 3.5 mM $MgCl_2$) providing amplification of wild-type (148 bp) and targeted allele (200 bp) fragments.

Targeted deletion of the *Id2* transcript was also confirmed by qRT-PCR from mRNA extracted from adult brain and liver, and from mouse embryonic fibroblasts of *Id2*+/+, *Id2*+/- and *Id2*-/- mice (liver mRNA is shown in Figure S2 D). Total RNA was isolated from tissues using Trizol reagent and qRT-PCR applied as described above using *Taqman* primers and probe specific for the detection of the *Id2* cDNA upstream of the exon 3 region (i.e. within exon 2). The 2.2 kb mutant protein product of *Neo-Exon3* contains no known functional domains as Exon 3 is wholly 3' UTR and contains no coding sequence, and thus is unlikely to form any functional protein. Absence of ID2 protein was confirmed by Western blot analysis of liver samples harvested at CT12 (Figure S2 E). These gene, mRNA and protein analyses, and the lack of any phenotype in the *Id2*+/- mice strongly suggest that our targeting strategy generated a null allele at *Id2*.

Western blots

Liver was homogenized in RIPA lysis buffer, plus 1% protease/phosphatase inhibitor cocktail (Sigma-Aldrich, St. Louis, MO), passed through a 25G 1.5 syringe and incubated at 4 ºC for 30 min. Samples were boiled in Laemmli sample buffer (Bio-Rad, Hercules, CA), and loaded at 40 or 80 μg protein per lane onto 12.5% SDS-polyacrylamide gels. Following separation at 125V for ~90 min, proteins were transferred onto nitrocellulose membranes, which were then blocked with 5% skim milk in Tris-buffered saline (TBS) with 0.05% Tween 20. Membranes were incubated with rabbit antisera against ID2 (C-20, 1:200, Santa Cruz Biotechnology, Santa Cruz, CA) or actin (1:1000, Cytoskeleton Inc, Denver, CO) overnight at 4 ºC. Immunoreactive bands were visualized using anti-rabbit secondary antiserum and ECL detection (Thermo Scientific, Waltham, MA).

Generation of *Id4*-/- mice

Id4 mutant mice were generated by homologous recombination as described in Yun *et al.* [36]: Exons 1 and 2 were replaced by the coding regions of *GFP* and the *neomycin-resistance* genes. Following transfection into embryonic stem cells, recombination events were confirmed by Southern blot (6.5 band) and PCR analyses. PCR analysis of mouse tails confirmed genotype of individual mice. Mice were established and maintained on a C57BL/6J background, and unlike the *Id2*-/- mice showed normal *Id4*-/- genotype productivity with heterozygote x heterozygote crosses.

Histological analysis of *Id2*-/- SCN

Brain tissue was collected for histological analysis from *Id2*+/+ (n = 6), *Id2*+/- (n = 6) and *Id2*-/- (n = 4) mice. Animals were killed by cervical dislocation and brains frozen on dry ice and stored in –80 °C freezer prior to sectioning on a cryostat. Coronal sections (16 μm) were collected on adhesive-coated slides (Fisher Scientific, Pittsburgh, PA) and treated with 4% para-formaldehyde for 5 min and two washes in 1xPBS. Slides were rehydrated in a descending series of alcohols, 100%, 95% and 70%, for 3 min each solution. Sections were briefly dipped in dd H_2O and immersed in Cresyl Violet (Sigma-Aldrich, St. Louis, MO) for 10-20 minutes depending on required intensity of staining. Sections were dehydrated in an ascending series of alcohols, 70%, 95% and 100%, 30 secs in each and cleared in xylene. Slides were coverslipped with Depex^{R} (VWR International, West Chester, PA).

Cresyl Violet (Nissl) stained sections were viewed under brightfield microscopy and images digitally captured (Diagnostic Instruments, Sterling Heights, MI). Three representative sections per animal were analyzed through the bilateral SCN region, representing rostral, medial and causal zones. Height and width (μm) and area (in μm$^{2)}$ of left and right SCN were measured using the

SPOT software program (Diagnostic Instruments) and the mean of these values calculated. Significant differences were determined by one-factor ANOVA (with False Discovery Rate Correction where interactions were apparent).

Locomotor activity monitoring and circadian phenotype analysis

F2 homozygous *Id2* mutants (n = 16) and their wild-type (n = 14) and heterozygous (n = 12) littermates were individually housed in cages equipped with a running-wheel and passive infrared motion detector, and activity was monitored for each animal. Mice were maintained on a 12:12 light:dark cycle (LD) for at least 10 days to establish stable entrainment and then transferred to DD for 30 days to measure free-running rhythms.

All experiments were conducted in accordance with the IACUCs at Dartmouth Medical School and the University of Notre Dame**.** Adult mice (≥ 3 months of age) were maintained in individual cages (29 x 11.5 x 13 cm) equipped with a running wheel (Actimetrics, Wilmette, IL) and a passive infrared movement detector (*Slimline PIR motion detector*, Smarthome, Irvine, CA). Food and water were available *ad libitum*. Temperature (22-25 ϒC) and humidity were maintained at a constant level. Mice were studied under a 12:12 LD cycle (150-400 lux, fluorescent lights: General Electric 36-W cool white), dim red light (< 1 lux) with lights on at 6 A.M. and off at 6 P.M., in constant dim red light DD (< 1 lux), and in constant light, LL (80 lux, neutral density filters, Lee Filters, Burbank, CA). White light was generated by. All mice received sterile water treated with antibiotic (Sulfamethoxalzole and trimethoprim oral suspension, Alpharma, Fort Lee, NJ, at a final dilution of 400 mg and 80 mg/L, respectively) and sterile food. Wheel-running and passive activity were monitored by a PC computer and using *Clocklab* hardware and software

(Actimetrics). Fourier (Fast Fourier Transformation) and χ^2 periodogram analyses were conducted on activity data using the *Clocklab* software. Lights off was defined as Zeitgeber time (ZT) 12. Circadian time (CT) 12 indicated activity onset under continuous darkness (DD). Period length under free-running conditions was determined using the periodogram analysis and determined from the slope of a hand-drawn line through activity onsets. The first 3-5 days in DD were removed from analysis to allow for stabilization of rhythms following transfer from LD to DD. In the re-entrainment and the delaying phase shift experiments, the light phase of the LD cycle was extended by 10 hr so that lights off changed from 6 P.M. to 4 A.M., and the lights on in the following LD cycle changed from 6 A.M. to 4 P.M. Assessment of stable entrainment was determined from actograms and from profiles of activity/24 hr. In the acute light pulse experiment, mice were entrained to a 12:12 LD cycle for 14 days and transferred to DD for 10 days of free-running activity. Mice were exposed to 30 min light (800 lux) starting at CT14 or CT16 (after initial 14 days in DD), and maintained for a further 10 days in DD. The magnitude of the phase delays were determined by drawing a line of best fit through 7-10 days of activity onsets immediately before the light treatment, and a second line through 7-10 days following the treatment. The phase shift was calculated as the difference between the actual onset of activity and that predicted by the pre-treatment line on the last day of treatment. Using actograms and profiles of mean activity/24hr, *alpha*, *phase angle* of activity onset relative to ZT12, *mean activity/24 hr*, *percentage activity during day* (ZT12-ZT0), and *percentage activity during estimated subjective day* were calculated from 10 days of continuous activity in LD and from the last 10 day segment in DD. Note that the data shown for mice treated with light at CT16 was collected from a different sub-population of mice generated and studied at the University of Notre Dame. For mice examined under LL (n = 8, 6, 11 for wild-type, heterozygote and *Id2*-/- mice, respectively), animals were individually housed

and entrained to a 12:12 LD for at least 14 days, and transferred to constant light for 30 days. Fourier and χ^2 periodogram analyses were undertaken on the third 10 day duration in LL. All other behavioral data shown was produced from a single population of mice studied at Dartmouth Medical School. Significant differences were determined by Fisher's Exact test ($p < 0.05$), Student's t-test (two-tailed distribution, $p < 0.05$), paired t-test ($p < 0.05$), and one-factor ANOVA, followed by post-hoc Dunnett's t-tests ($p < 0.05$). Unless stated otherwise, the statistical test refers to a one-factor ANOVA.

Transfection and luciferase studies

NIH3T3 cells were seeded (3×10^4 cells per well) in 24-well dishes in DMEM with 5% FBS. Cells were transfected with Lipofectamine-Plus (Invitrogen) according to the manufacturer's instructions, together with 500 ng total per well of pcDNA3 or pCMV expression plasmids with the indicated inserts (125 ng of each), and either *mPer1* or *AVP* promoter construct in the pGL3 basic reporter plasmid (10 ng) [37, 38]. Transfection efficiency was assessed by cotransfection of cells with *Renilla* pRL-CMV (0.5 ng, Promega) and/or β-galactocidase-CMV (25 ng) internal control reporter plasmids. Expression vectors encoded hamster BMAL1, mouse CLOCK, mCRY1, mPER1, and human ID1, ID2, ID3 and ID4. An assay of ID4 effect was repeated using a human *Id4* pMSCV-IRES plasmid. In the dose dependent experiment, *Id2* plasmid was added at quantities of 500, 250, 125, 63, 32, 16, 8 ng per well. The total concentration per well was adjusted to 500 ng per well with pcDNA3 and pCMV empty vector (Invitrogen). Cells were lysed 48 hr following transfection with 120 μl Passive Lysis Buffer (Promega) and firefly and *Renilla* luciferase activities assayed with Dual Reporter Assay reagent (Promega) and by luminometry, and β-galactocidase activity measured using Z-buffer reagent and by measurement of optical density by spectrophotometry. For

each sample, luciferase activity was corrected for transfection efficiency by dividing the firefly activity value by the *Renilla* or β-galactocidase activity value. Percentage inhibition was determined from five independent experiments and 100% inhibition was equal to spontaneous activity background levels produced with empty expression vector. The *mPer1* or *AVP* promoter vectors and the expression vectors containing *Bmal1*, *Clock*, *mCry1* and *mPer1* were generously provided by S. M. Reppert and C. J. Weitz [37, 38]. The *Id* expression plasmids were prepared from cDNA containing full-length coding regions and by ligation into either pcDNA3 (*Id2*; Accession number M97796; Invitogen, Carlsbad, CA) or pCMV (*Id1*, *Id3*) [39] (Stratagene, Carlsbad, CA) vector. Full-length coding sequence of human *Id4* in pcDNA3 was kindly provided by M. Rigolet (Accession number Y07958), and the *Id4* gene was subcloned into an alternative pMSCV-IRES vector.

Supplemental References

1. Storch, K.F., Lipan, O., Leykin, I., Viswanathan, N., Davis, F.C., Wong, W., and Weitz, C.J. (2002). Extensive and divergent circadian gene expression in liver and heart. Nature *417*, 78-83.
2. Panda, S., Antoch, M.P., Miller, B., Su, A., Schook, A., Straume, M., Schultz, P., Kay, S., Takahashi, J., and Hogenesch, J.B. (2002). Coordinated transcription of key pathways in the mouse by the circadian clock. Cell *109*, 307-320.
3. Akhtar, R.A., Reddy, A., Maywood, E.S., Clayton, J.D., King, V., Smith, A.G., Gant, T.W., Hastings, M.H., and Kyriacou, C.P. (2002). Circadian transcriptional cycling in the mouse

liver, as revealed by cDNA microarrays, is driven by the suprachiasmatic nucleus. Current Biology *12*, 540-550.

4. Duffield, G.E., Best, J.D., Meurers, B.H., Bittner, A., Loros, J.J., and Dunlap, J.C. (2002). Circadian programs of transcriptional activation, signaling, and protein turnover revealed by microarray analysis of mammalian cells. Current Biology *12*, 551-557.

5. Humphries, A., Klein, D., Baler, R., and Carter, D.A. (2002). cDNA array analysis of pineal gene expression reveals circadian rhythmicity of the dominant negative helix-loop-helix protein-encoding gene, *Id-1*. J Neuroendocrinol *14*, 101-108.

6. Ueda, H.R., Chen, W., Adachi, A., Wakamatsu, H., Hayashi, S., Takasugi, T., Nagano, M., Nakahama, K., Suzuki, Y., Sugano, S., Iino, M., Shigeyoshi, Y., and Hashimoto, S. (2002). A transcription factor response element for gene expression during circadian night. Nature *418*, 534 - 539.

7. Menger, G.J., Lu, K., Thomas, T., Cassone, V.M., and Earnest, D.J. (2005). Circadian profiling of the transcriptome in immortalized rat SCN cells. Physiol Genomics *21*, 370-381.

8. Duffield, G.E. (2003). DNA microarray analyses of circadian timing: the genomic basis of biological time. J Neuroendocrinol. *15*, 991-1002.

9. Rudic, R.D., McNamara, P., Reilly, D., Grosser, T., Curtis, A.M., Price, T.S., Panda, S., Hogenesch, J.B., and FitzGerald, G.A. (2005). Bioinformatic analysis of circadian gene oscillation in mouse aorta. Circulation *112*, 2716-2724.

10. Hughes, M., DeHaro, L., Pulivarthy, S.R., Gu, J., Hayes, K., Panda, S., and Hogenesch, J.B. (2007). High-resolution Time Course Analysis of Gene Expression from Pituitary. Cold Spring Harbor Symposia on Quantitative Biology *72*, 381-386.

11. Sato, T., Panda, S., Kay, S., and Hogenesch, J.B. (2003). DNA Arrays: Applications and Implications for Circadian Biology. J Biol Rhythms *18*, 96-105.

12. Yokota, Y., Mansouri, A., Mori, S., Sugawara, S., Adachi, S., Nishikawa, S.-I., and Gruss, P. (1999). Development of peripheral lymphoid organs and natural killer cells depends on the helix-loop-helix inhibitor Id2. Nature *397*, 702-706.

13. Kim, J.K., Takeuchi, M., and Yokota, Y. (2004). Impairment of intestinal intraepithelial lymphocytes in Id2 deficient mice. Gut *53*, 480-486.

14. Kusunoki, T., Sugai, M., Katakai, T., Omatsu, Y., Iyoda, T., Inaba, K., Nakahata, T., Shimizu, A., and Yokota, Y. (2003). TH2 dominance and defective development of a CD8+ dendritic cell subset in Id2-deficient mice. J Allergy Clin Immunol. J. Allergy Clin Immunol *111*, 136-142.

15. Sugai, M., Gonda, H., Kusunoki, T., Katakai, T., Yokota, Y., and Shimizu, A. (2003). Essential role of Id2 in negative regulation of IgE class switching. Nat Immunol *4*, 25-30.

16. Yokota, Y., Mori, S., Narumi, O., and Kitajima, K. (2001). *In vivo* function of a differentiation inhibitor, Id2. IUBMB Life *51*, 207-214.

17. Moskowitz, I.P., Kim, J.B., Moore, M.L., Wolf, C.M., Peterson, M.A., Shendure, J., Nobrega, M.A., Yokota, Y., Berul, C., Izumo, S., Seidman, J.G., and Seidman, C.E. (2007). A molecular pathway including Id2, Tbx5, and Nkx2-5 required for cardiac conduction system development. Cell *129*, 1365-1376.

18. Aoki, Y., Mori, S., Kitajima, K., Yokoyama, O., Kanamaru, H., Okada, K., and Yokota, Y. (2004). Id2 haploinsufficiency in mice leads to congenital hydronephrosis resembling that in humans. Genes Cells *9*, 1287-1296.

19. Park, K.W., Waki, H., Villanueva, C.J., Monticelli, L.A., Hong, C., Kang, S., MacDougald, O.A., Goldrath, A.W., and Tontonoz, P. (2008). Inhibitor of DNA binding 2 is a small molecule-inducible modulator of peroxisome proliferator-activated receptor-gamma expression and adipocyte differentiation. Mol Endocrinol *22*, 2038-2048.

20. Shearman, L.P., and Weaver, D.R. (1999). Photic induction of *Period* gene expression is reduced in *Clock* mutant mice. NeuroReport *10*, 613-618.

21. Jung, H., Choe, Y., Kim, H., Park, N., Son, G.H., Khang, I., and Kim, K. (2003). Involvement of CLOCK:BMAL1 heterodimer in serum-responsive *mPer1* induction. Neuroreport *14*, 15-19.

22. Reppert, S.M., and Weaver, D.R. (2001). Molecular analysis of mammalian circadian rhythms. Annu Rev Physiol. *63*, 647-676.

23. Travnickova-Bendova, Z., Cermakian, N., Reppert, S.M., and Sassone-Corsi, P. (2002). Bimodal regulation of mPeriod promoters by CREB-dependent signaling and CLOCK/BMAL1 activity. Proc Natl Acad Sci U S A *99*, 7728-7733.

24. Tischkau, S.A., Mitchell, J.W., Tyan, S.H., Buchanan, G.F., and Gillette, M.U. (2003). Ca2+/cAMP response element-binding protein (CREB)-dependent activation of *Per1* is required for light-induced signaling in the suprachiasmatic nucleus circadian clock. J Biol Chem *278*, 718-723.

25. Shim, H.S., Kim, H., Lee, J., Son, G.H., Cho, S., Oh, T.H., Kang, S.H., Seen, D.S., Lee, K.H., and Kim, K. (2007). Rapid activation of CLOCK by Ca2+-dependent protein kinase C mediates resetting of the mammalian circadian clock. EMBO Rep *8*, 366-371.

26. Muñoz, M., S.N., P., Hankins, M.W., and Foster, R.G. (2005). Long-term constant light induces constitutive elevated expression of mPER2 protein in the murine SCN: a molecular basis for Aschoff's rule? J Biol Rhythms *20*, 3-14.

27. Wang, K.R., Nemoto, T., and Yokota, Y. (2007). RFX1 mediates the serum-induced immediate early response of *Id2* gene expression. J Biol Chem *282*, 26167-26177.

28. Shigeyoshi, Y., Taguchi, K., Yamamoto, S., Takeida, S., Yan, L., Tei, H., Moriya, S., Shibata, S., Loros, J.J., Dunlap, J.C., and Okamura, H. (1997). Light-induced resetting of a mammalian circadian clock is associated with rapid induction of the *mPer1* transcript. Cell *91*, 1043 - 1053.

29. Albrecht, U., Sun, Z., Eichele, G., and Lee, C. (1997). A differential response of two putative mammalian circadian regulators, *mper1* and *mper2,* to light. Cell *91*, 1055 - 1064.

30. Duffield, G.E., McNulty, S., and Ebling, F.J. (1999). Anatomical and functional characterisation of a dopaminergic system in the suprachiasmatic nucleus of the neonatal Siberian hamster. J Comp Neurol *408*, 73-96.

31. Rusak, B., McNaughton, L., Robertson, H.A., and Hunt, S.P. (1992). Circadian variation in photic regulation of immediate-early gene mRNAs in rat suprachiasmatic nucleus cells. Brain Res Mol Brain Res *14*, 124-130.

32. Peirson, S.N., Butler, J.N., Duffield, G.E., Takher, S., Sharma, P., and Foster, R.G. (2006). Comparison of clock gene expression in SCN, retina, heart, and liver of mice. Biochem Biophys Res Commun *351*, 800-807.

33. Peirson, S.N., Butler, J.N., and Foster, R.G. (2003). Experimental validation of novel and conventional approaches to quantitative real-time PCR data analysis. Nucleic Acids Res *31*, e73.

34. Mantani, A., Hernandez, M.C., Kuo, W.L., and Israel, M.A. (1998). The mouse Id2 and Id4 genes: structural organization and chromosomal localization. Gene *222*, 229-235.

35. Van Gelder, R.N., and Hogenesch, J.B. (2004). Clean thoughts about dirty genes. J Biol Rhythms *19*, 3-9.

36. Yun, K., Mantani, A., Garel, S., Rubenstein, J., and Israel, M.A. (2004). Id4 regulates neural progenitor proliferation and differentiation *in vivo*. Development *131*, 5441-5448.

37. Gekakis, N., Stankis, D., Nguyen, H.B., Davis, F.C., Wilsbacher, L.D., King, D.P., Takahashi, J.S., and Weitz, C.J. (1998). Role of the CLOCK protein in the mammalian circadian mechanism. Science *280*, 1564-1569.

38. Kume, K., Zylka, M.J., Sriram, S., Shearman, L.P., Weaver, D.R., Jin, X., Maywood, E.S., Hastings, M.H., and Reppert, S.M. (1999). mCRY1 and mCRY2 are essential components of the negative limb of the circadian clock feedback loop. Cell *98*, 193-205.

39. Lasorella, A., Iavarone, A., and Israel, M.A. (1996). Id2 Specifically Alters Regulation of the Cell Cycle by Tumor Suppressor Proteins. Molecular and Cellular Biology *16*, 2570-2578.
